# Correction: Social determinants of food group consumption based on Mediterranean diet pyramid: A cross-sectional study of university students

**DOI:** 10.1371/journal.pone.0238270

**Published:** 2020-08-21

**Authors:** Roberto Martinez-Lacoba, Isabel Pardo-Garcia, Elisa Amo-Saus, Francisco Escribano-Sotos

The ORCID iDs are missing for the second, third, and fourth authors. Please see the authors’ respective ORCID iDs here:

Author Isabel Pardo-Garcia’s ORCID iD is: 0000-0003-4391-6011 (https://orcid.org/0000-0003-4391-6011).

Author Elisa Amo-Saus’s ORCID iD is: 0000-0002-2100-9325 (https://orcid.org/0000-0002-2100-9325).

Author Francisco Escribano-Sotos’s ORCID iD is: 0000-0002-2747-6725 (https://orcid.org/0000-0002-2747-6725).
