# Supplementary material for: Social determinants of food group consumption based on Mediterranean diet pyramid: A cross-sectional study of university students
Source: PLoS One. 2020 Jan 30;15(1):e0227620. doi: 10.1371/journal.pone.0227620 (PMC6992217; doi:10.1371/journal.pone.0227620)
Supplement: S1 Supporting information [file pone.0227620.s001.docx]

**Appendix A**

# Calculation of variables

## Food frequency questionnaire: individual foods

All collected foods are showed in Table A.

## Food groups

Food groups and recommended consumption are based on the Mediterranean diet pyramid (Bach-Faig et al., 2011). Foods and food groups are detailed in Table B. We also collected information about the consumption of fast food, precooked food and alcoholic beverages. The recommended intake of alcoholic beverages is based on another study (Sofi, Macchi, Abbate, Gensini, & Casini, 2013), and it was calculated by using alcohol units as follows: millilitres · serving · 0.1 · volume of alcohol.

# Missing data analysis

We included the following variables in the imputation model: gender, SES, location of the family home, whether the participant cooked for themselves during the academic year, and the degree course (Health or Social Sciences). The variables were coded using a dummy coding. Table D includes the percentage of missing values for each variable. All food groups have the same percentage of missing values. It is explained because we did not calculate the food consumption of the subjects who presented extreme or missing values in any variable. We performed two multiple imputation analyses with 5 and 30 subsets (number of iterations=20). Density plots of the food groups after imputation of values are showed in Figs B and C. The results from the linear regression with complete-case analysis and multiple imputation analyses (both, m=5 and m=30 subsets) are showed in the following section.

# Multiple regression outputs of complete-case analysis, and multiple imputation analyses (m=5 and m=30 subsets).

## Summary of results

Table G shows the summary of the regression results with complete-case and imputed data analysis.

## Description of output

We show the results from all regressions, where:

i) Complete-case analysis: a) Estimate = β coefficient; b) Standardized = standardized β coefficient; c) Std. Error = Standard Error; d) t value = t value;
 e) Pr ( > | t | ) = p-value.

ii) Multiple imputation analyses: a) estimate = β coefficient; b) std.error = Standard Error; c) statistic = t value; df = residual degrees of freedom; p.value = p-value.

The independent variables of the models are: genderdummy = gender; hiseidummy1 = SES (1); hiseidummy2 = SES (2); familyhome1 = Family home (1); familyhome2 = Family home (2); cook1 = He/she Cooks for him/herself; degree1= Degree; gender_hisei1 = Gender x SES (1); gender_hisei2 = Gender x SES (2); gender_family1 = Gender x Family home (1); gender_family2 = Gender x Family home (2); gender_cook1 = Gender x He/she cooks for him/herself; gender_degree1=Gender x Degree

Note that “**.**” **=** p<0.10; “*” = p<0.05

## Complete-case analysis

DAIRY PRODUCTS

Coefficients:

Estimate Standardized Std. Error t value Pr(>|t|)

(Intercept) 2.8082 0.0000 0.1326 21.18 <2e-16 ***

genderdummy 0.0888 0.0434 0.1326 0.67 0.50

hiseidummy1 -0.0741 -0.0544 0.0611 -1.21 0.23

hiseidummy2 -0.1312 -0.0472 0.1183 -1.11 0.27

familyhome1 0.0955 0.0514 0.0799 1.20 0.23

familyhome2 0.0813 0.0350 0.1014 0.80 0.42

cook1 -0.0451 -0.0203 0.0985 -0.46 0.65

degree1 -0.0314 -0.0133 0.1102 -0.29 0.78

gender_hisei1 -0.0643 -0.0476 0.0611 -1.05 0.29

gender_hisei2 -0.1916 -0.0722 0.1183 -1.62 0.11

gender_family1 0.1091 0.0653 0.0799 1.37 0.17

gender_family2 0.0811 0.0369 0.1014 0.80 0.42

gender_cook1 -0.0208 -0.0103 0.0985 -0.21 0.83

gender_degree1 -0.0745 -0.0368 0.1102 -0.68 0.50

OLIVES NUTS SEEDS

Coefficients:

Estimate Standardized Std. Error t value Pr(>|t|)

(Intercept) 0.36567 0.00000 0.03137 11.66 <2e-16 ***

genderdummy 0.06118 0.12689 0.03137 1.95 0.052 .

hiseidummy1 -0.00570 -0.01775 0.01446 -0.39 0.693

hiseidummy2 0.00314 0.00479 0.02799 0.11 0.911

familyhome1 -0.03051 -0.06963 0.01890 -1.61 0.107

familyhome2 0.03309 0.06045 0.02398 1.38 0.168

cook1 -0.03585 -0.06848 0.02330 -1.54 0.124

degree1 0.03258 0.05842 0.02607 1.25 0.212

gender_hisei1 -0.00517 -0.01624 0.01446 -0.36 0.721

gender_hisei2 0.00549 0.00877 0.02799 0.20 0.845

gender_family1 0.01423 0.03612 0.01890 0.75 0.452

gender_family2 0.02285 0.04410 0.02398 0.95 0.341

gender_cook1 0.02333 0.04893 0.02330 1.00 0.317

gender_degree1 0.03761 0.07875 0.02607 1.44 0.150

HERBS SPICES GARLIC ONIONS

Coefficients:

Estimate Standardized Std. Error t value Pr(>|t|)

(Intercept) 0.52562 0.00000 0.04882 10.77 <2e-16 ***

genderdummy 0.04803 0.06433 0.04882 0.98 0.33

hiseidummy1 -0.03003 -0.06035 0.02250 -1.33 0.18

hiseidummy2 -0.03327 -0.03281 0.04356 -0.76 0.45

familyhome1 0.04521 0.06662 0.02941 1.54 0.12

familyhome2 0.04961 0.05852 0.03732 1.33 0.18

cook1 0.01027 0.01266 0.03625 0.28 0.78

degree1 0.00179 0.00207 0.04056 0.04 0.96

gender_hisei1 0.02140 0.04341 0.02250 0.95 0.34

gender_hisei2 0.01618 0.01669 0.04356 0.37 0.71

gender_family1 -0.01524 -0.02498 0.02941 -0.52 0.60

gender_family2 0.04469 0.05570 0.03732 1.20 0.23

gender_cook1 -0.00220 -0.00298 0.03625 -0.06 0.95

gender_degree1 -0.00238 -0.00322 0.04056 -0.06 0.95

FRUITS

Coefficients:

Estimate Standardized Std. Error t value Pr(>|t|)

(Intercept) 2.940472 0.000000 0.155016 18.97 <2e-16 ***

genderdummy -0.107965 -0.045569 0.155016 -0.70 0.49

hiseidummy1 -0.046830 -0.029664 0.071436 -0.66 0.51

hiseidummy2 -0.127775 -0.039704 0.138307 -0.92 0.36

familyhome1 -0.023311 -0.010826 0.093379 -0.25 0.80

familyhome2 0.070679 0.026273 0.118508 0.60 0.55

cook1 0.100327 0.039000 0.115120 0.87 0.38

degree1 0.097623 0.035628 0.128797 0.76 0.45

gender_hisei1 -0.018367 -0.011740 0.071436 -0.26 0.80

gender_hisei2 0.000457 0.000148 0.138307 0.00 1.00

gender_family1 0.056297 0.029078 0.093379 0.60 0.55

gender_family2 -0.128390 -0.050430 0.118508 -1.08 0.28

gender_cook1 -0.057068 -0.024364 0.115120 -0.50 0.62

gender_degree1 0.164311 0.070018 0.128797 1.28 0.20

VEGETABLES

Coefficients:

Estimate Standardized Std. Error t value Pr(>|t|)

(Intercept) 2.19544 0.00000 0.15782 13.91 <2e-16 ***

genderdummy -0.24360 -0.10041 0.15782 -1.54 0.123

hiseidummy1 0.09181 0.05680 0.07273 1.26 0.207

hiseidummy2 0.14776 0.04484 0.14081 1.05 0.294

familyhome1 0.13159 0.05969 0.09507 1.38 0.167

familyhome2 0.18533 0.06728 0.12065 1.54 0.125

cook1 0.17253 0.06550 0.11720 1.47 0.142

degree1 0.07099 0.02530 0.13112 0.54 0.588

gender_hisei1 0.06756 0.04217 0.07273 0.93 0.353

gender_hisei2 0.04150 0.01318 0.14081 0.29 0.768

gender_family1 0.01001 0.00505 0.09507 0.11 0.916

gender_family2 0.08173 0.03135 0.12065 0.68 0.498

gender_cook1 0.05665 0.02362 0.11720 0.48 0.629

gender_degree1 -0.25031 -0.10417 0.13112 -1.91 0.057 .

OLIVE OIL

Coefficients:

Estimate Standardized Std. Error t value Pr(>|t|)

(Intercept) 1.330981 0.000000 0.058745 22.66 < 2e-16 ***

genderdummy 0.000786 0.000861 0.058745 0.01 0.99

hiseidummy1 0.004635 0.007619 0.027072 0.17 0.86

hiseidummy2 0.071640 0.057763 0.052413 1.37 0.17

familyhome1 -0.037429 -0.045104 0.035387 -1.06 0.29

familyhome2 0.033038 0.031867 0.044910 0.74 0.46

cook1 0.024087 0.024296 0.043626 0.55 0.58

degree1 0.208293 0.197246 0.048809 4.27 0.000023 ***

gender_hisei1 -0.025845 -0.042864 0.027072 -0.95 0.34

gender_hisei2 0.048232 0.040687 0.052413 0.92 0.36

gender_family1 -0.004370 -0.005857 0.035387 -0.12 0.90

gender_family2 -0.017113 -0.017441 0.044910 -0.38 0.70

gender_cook1 -0.027029 -0.029942 0.043626 -0.62 0.54

gender_degree1 0.056101 0.062031 0.048809 1.15 0.25

BREAD PASTA RICES

Coefficients:

Estimate Standardized Std. Error t value Pr(>|t|)

(Intercept) 2.369308 0.000000 0.094024 25.20 <2e-16 ***

genderdummy 0.203790 0.139688 0.094024 2.17 0.0306 *

hiseidummy1 0.006968 0.007168 0.043329 0.16 0.8723

hiseidummy2 -0.000273 -0.000138 0.083890 0.00 0.9974

familyhome1 -0.070705 -0.053328 0.056639 -1.25 0.2124

familyhome2 0.054953 0.033175 0.071881 0.76 0.4449

cook1 -0.137207 -0.086621 0.069826 -1.96 0.0499 *

degree1 0.219044 0.129826 0.078122 2.80 0.0052 **

gender_hisei1 0.113706 0.118034 0.043329 2.62 0.0089 **

gender_hisei2 0.077997 0.041181 0.083890 0.93 0.3529

gender_family1 0.011504 0.009650 0.056639 0.20 0.8391

gender_family2 -0.121339 -0.077402 0.071881 -1.69 0.0919 .

gender_cook1 0.022092 0.015317 0.069826 0.32 0.7518

gender_degree1 0.024266 0.016793 0.078122 0.31 0.7562

POTATOES

Coefficients:

Estimate Standardized Std. Error t value Pr(>|t|)

(Intercept) 1.40256 0.00000 0.09818 14.28 <2e-16 ***

genderdummy -0.01297 -0.00862 0.09818 -0.13 0.895

hiseidummy1 0.02774 0.02765 0.04525 0.61 0.540

hiseidummy2 -0.05299 -0.02592 0.08760 -0.60 0.545

familyhome1 -0.04282 -0.03130 0.05914 -0.72 0.469

familyhome2 0.07750 0.04534 0.07506 1.03 0.302

cook1 0.06316 0.03864 0.07291 0.87 0.387

degree1 0.07378 0.04238 0.08158 0.90 0.366

gender_hisei1 -0.00943 -0.00949 0.04525 -0.21 0.835

gender_hisei2 -0.06316 -0.03232 0.08760 -0.72 0.471

gender_family1 0.00128 0.00104 0.05914 0.02 0.983

gender_family2 0.02866 0.01772 0.07506 0.38 0.703

gender_cook1 -0.17187 -0.11549 0.07291 -2.36 0.019 *

gender_degree1 0.09505 0.06375 0.08158 1.17 0.244

RED MEAT AND PROCESSED MEAT

Coefficients:

Estimate Standardized Std. Error t value Pr(>|t|)

(Intercept) 13.36953 0.00000 0.59459 22.49 <2e-16 ***

genderdummy 1.10891 0.12043 0.59459 1.86 0.063 .

hiseidummy1 0.50878 0.08292 0.27401 1.86 0.064 .

hiseidummy2 -0.62763 -0.05018 0.53050 -1.18 0.237

familyhome1 -0.32884 -0.03930 0.35817 -0.92 0.359

familyhome2 -0.47384 -0.04532 0.45456 -1.04 0.298

cook1 -0.19688 -0.01969 0.44157 -0.45 0.656

degree1 0.03870 0.00363 0.49403 0.08 0.938

gender_hisei1 0.32155 0.05288 0.27401 1.17 0.241

gender_hisei2 0.19467 0.01628 0.53050 0.37 0.714

gender_family1 0.39183 0.05207 0.35817 1.09 0.274

gender_family2 0.80455 0.08131 0.45456 1.77 0.077 .

gender_cook1 0.30389 0.03338 0.44157 0.69 0.492

gender_degree1 0.65308 0.07161 0.49403 1.32 0.187

SWEETS

Coefficients:

Estimate Standardized Std. Error t value Pr(>|t|)

(Intercept) 6.06887 0.00000 0.41619 14.58 <2e-16 ***

genderdummy 0.42247 0.06601 0.41619 1.02 0.310

hiseidummy1 -0.16587 -0.03889 0.19179 -0.86 0.387

hiseidummy2 -0.05397 -0.00621 0.37133 -0.15 0.884

familyhome1 -0.27858 -0.04789 0.25070 -1.11 0.267

familyhome2 0.59619 0.08204 0.31817 1.87 0.061 .

cook1 -0.56027 -0.08062 0.30907 -1.81 0.070 .

degree1 -0.24049 -0.03249 0.34579 -0.70 0.487

gender_hisei1 -0.13615 -0.03221 0.19179 -0.71 0.478

gender_hisei2 0.19388 0.02333 0.37133 0.52 0.602

gender_family1 0.26744 0.05113 0.25070 1.07 0.287

gender_family2 0.32828 0.04773 0.31817 1.03 0.303

gender_cook1 0.05998 0.00948 0.30907 0.19 0.846

gender_degree1 0.13709 0.02163 0.34579 0.40 0.692

WHITE MEAT

Coefficients:

Estimate Standardized Std. Error t value Pr(>|t|)

(Intercept) 3.6702 0.0000 0.2202 16.67 <2e-16 ***

genderdummy 0.3160 0.0932 0.2202 1.44 0.152

hiseidummy1 0.0622 0.0275 0.1015 0.61 0.540

hiseidummy2 -0.2817 -0.0611 0.1964 -1.43 0.152

familyhome1 -0.2421 -0.0785 0.1326 -1.83 0.068 .

familyhome2 -0.1892 -0.0491 0.1683 -1.12 0.262

cook1 0.1994 0.0541 0.1635 1.22 0.223

degree1 -0.1237 -0.0315 0.1829 -0.68 0.499

gender_hisei1 0.0371 0.0165 0.1015 0.37 0.715

gender_hisei2 -0.1750 -0.0397 0.1964 -0.89 0.373

gender_family1 0.0440 0.0159 0.1326 0.33 0.740

gender_family2 0.0529 0.0145 0.1683 0.31 0.753

gender_cook1 0.3010 0.0897 0.1635 1.84 0.066 .

gender_degree1 0.1877 0.0559 0.1829 1.03 0.305

FISH SEAFOOD

Coefficients:

Estimate Standardized Std. Error t value Pr(>|t|)

(Intercept) 5.521914 0.000000 0.289201 19.09 <2e-16 ***

genderdummy -0.289640 -0.065751 0.289201 -1.00 0.32

hiseidummy1 0.098756 0.033646 0.133273 0.74 0.46

hiseidummy2 -0.084507 -0.014124 0.258028 -0.33 0.74

familyhome1 -0.100132 -0.025012 0.174211 -0.57 0.57

familyhome2 0.019730 0.003945 0.221091 0.09 0.93

cook1 -0.282712 -0.059110 0.214770 -1.32 0.19

degree1 -0.040568 -0.007963 0.240287 -0.17 0.87

gender_hisei1 -0.125372 -0.043101 0.133273 -0.94 0.35

gender_hisei2 -0.348857 -0.061000 0.258028 -1.35 0.18

gender_family1 0.002287 0.000635 0.174211 0.01 0.99

gender_family2 0.127468 0.026929 0.221091 0.58 0.56

gender_cook1 -0.096118 -0.022071 0.214770 -0.45 0.65

gender_degree1 0.050068 0.011475 0.240287 0.21 0.84

EGGS

Coefficients:

Estimate Standardized Std. Error t value Pr(>|t|)

(Intercept) 3.2717 0.0000 0.2087 15.68 < 2e-16 ***

genderdummy 0.8936 0.2723 0.2087 4.28 0.000022 ***

hiseidummy1 0.0690 0.0316 0.0962 0.72 0.473

hiseidummy2 0.2296 0.0515 0.1862 1.23 0.218

familyhome1 -0.0645 -0.0216 0.1257 -0.51 0.608

familyhome2 -0.1325 -0.0356 0.1595 -0.83 0.407

cook1 0.3782 0.1061 0.1550 2.44 0.015 *

degree1 0.0869 0.0229 0.1734 0.50 0.617

gender_hisei1 0.0453 0.0209 0.0962 0.47 0.638

gender_hisei2 0.2848 0.0669 0.1862 1.53 0.127

gender_family1 0.1337 0.0499 0.1257 1.06 0.288

gender_family2 -0.2736 -0.0776 0.1595 -1.71 0.087 .

gender_cook1 0.0796 0.0245 0.1550 0.51 0.608

gender_degree1 0.0940 0.0289 0.1734 0.54 0.588

LEGUMES

Coefficients:

Estimate Standardized Std. Error t value Pr(>|t|)

(Intercept) 3.5330 0.0000 0.1799 19.63 <2e-16 ***

genderdummy 0.3877 0.1394 0.1799 2.15 0.032 *

hiseidummy1 0.0573 0.0309 0.0829 0.69 0.490

hiseidummy2 -0.1791 -0.0474 0.1605 -1.12 0.265

familyhome1 -0.1693 -0.0670 0.1084 -1.56 0.119

familyhome2 0.1103 0.0349 0.1376 0.80 0.423

cook1 -0.1172 -0.0388 0.1336 -0.88 0.381

degree1 0.0876 0.0272 0.1495 0.59 0.558

gender_hisei1 0.1679 0.0914 0.0829 2.02 0.043 *

gender_hisei2 -0.1303 -0.0361 0.1605 -0.81 0.417

gender_family1 -0.0794 -0.0349 0.1084 -0.73 0.464

gender_family2 -0.0380 -0.0127 0.1376 -0.28 0.782

gender_cook1 0.3331 0.1211 0.1336 2.49 0.013 *

gender_degree1 -0.0677 -0.0246 0.1495 -0.45 0.651

ALCOHOLIC DRINKS

Coefficients:

Estimate Standardized Std. Error t value Pr(>|t|)

(Intercept) 0.57912 0.00000 0.09083 6.38 3.7e-10 ***

genderdummy 0.07974 0.05613 0.09083 0.88 0.38035

hiseidummy1 -0.06346 -0.06704 0.04186 -1.52 0.13004

hiseidummy2 0.06097 0.03160 0.08104 0.75 0.45210

familyhome1 0.00813 0.00630 0.05471 0.15 0.88187

familyhome2 0.04632 0.02872 0.06944 0.67 0.50495

cook1 0.01525 0.00989 0.06745 0.23 0.82118

degree1 -0.26547 -0.16159 0.07547 -3.52 0.00047 ***

gender_hisei1 -0.09028 -0.09625 0.04186 -2.16 0.03142 *

gender_hisei2 -0.05186 -0.02812 0.08104 -0.64 0.52247

gender_family1 0.00859 0.00740 0.05471 0.16 0.87525

gender_family2 0.06225 0.04078 0.06944 0.90 0.37033

gender_cook1 -0.05100 -0.03632 0.06745 -0.76 0.44986

gender_degree1 -0.08589 -0.06104 0.07547 -1.14 0.25554

FAST FOOD

Coefficients:

Estimate Standardized Std. Error t value Pr(>|t|)

(Intercept) 0.65834 0.00000 0.02796 23.54 <2e-16 ***

genderdummy 0.07868 0.18231 0.02796 2.81 0.0051 **

hiseidummy1 0.02264 0.07872 0.01289 1.76 0.0795 .

hiseidummy2 0.00150 0.00255 0.02495 0.06 0.9522

familyhome1 -0.02301 -0.05866 0.01684 -1.37 0.1725

familyhome2 0.00371 0.00756 0.02138 0.17 0.8624

cook1 -0.00476 -0.01015 0.02077 -0.23 0.8189

degree1 -0.01070 -0.02144 0.02323 -0.46 0.6453

gender_hisei1 0.01383 0.04852 0.01289 1.07 0.2837

gender_hisei2 -0.00255 -0.00456 0.02495 -0.10 0.9185

gender_family1 -0.03494 -0.09908 0.01684 -2.07 0.0385 *

gender_family2 -0.00743 -0.01602 0.02138 -0.35 0.7283

gender_cook1 -0.00919 -0.02154 0.02077 -0.44 0.6582

gender_degree1 0.00302 0.00706 0.02323 0.13 0.8967

PRECOOKED FOOD

Coefficients:

Estimate Standardized Std. Error t value Pr(>|t|)

(Intercept) 1.01732 0.00000 0.05774 17.62 <2e-16 ***

genderdummy -0.04808 -0.05443 0.05774 -0.83 0.405

hiseidummy1 0.03547 0.06026 0.02661 1.33 0.183

hiseidummy2 0.01275 0.01062 0.05152 0.25 0.805

familyhome1 -0.01806 -0.02250 0.03478 -0.52 0.604

familyhome2 0.01736 0.01730 0.04414 0.39 0.694

cook1 0.00934 0.00974 0.04288 0.22 0.828

degree1 -0.06551 -0.06413 0.04797 -1.37 0.173

gender_hisei1 -0.02304 -0.03950 0.02661 -0.87 0.387

gender_hisei2 -0.11285 -0.09841 0.05152 -2.19 0.029 *

gender_family1 0.02543 0.03523 0.03478 0.73 0.465

gender_family2 -0.01236 -0.01302 0.04414 -0.28 0.780

gender_cook1 0.00462 0.00529 0.04288 0.11 0.914

gender_degree1 0.02972 0.03397 0.04797 0.62 0.536

## Imputed data (m=5)

DAIRY PRODUCTS

estimate std.error statistic df p.value

(Intercept) 2.84907 0.12962 21.98072 50.70794 0.00000

genderdummy 0.16151 0.12560 1.28591 72.54666 0.19929

hiseidummy1 -0.06349 0.06375 -0.99597 23.61118 0.31993

hiseidummy2 -0.15842 0.12235 -1.29488 28.31252 0.19618

familyhome1 0.10271 0.08556 1.20040 29.71904 0.23076

familyhome2 0.05185 0.09577 0.54137 56.88348 0.58858

cook1 -0.01344 0.11032 -0.12187 17.19910 0.90307

degree1 0.00005 0.10150 0.00048 62.85960 0.99962

gender_hisei1 -0.05934 0.06357 -0.93347 23.99026 0.35119

gender_hisei2 -0.09288 0.10145 -0.91557 364.71494 0.36050

gender_family1 0.10419 0.08275 1.25906 38.30594 0.20881

gender_family2 0.05404 0.10025 0.53911 36.95445 0.59014

gender_cook1 0.00401 0.10776 0.03718 19.14884 0.97036

gender_degree1 -0.02778 0.09218 -0.30136 361.87584 0.76331

OLIVES NUTS SEEDS

estimate std.error statistic df p.value

(Intercept) 0.36457 0.03202 11.38639 32.63425 0.00000

genderdummy 0.05381 0.02678 2.00927 502.78986 0.04504*

hiseidummy1 -0.00736 0.01716 -0.42869 13.02485 0.66833

hiseidummy2 -0.00229 0.03092 -0.07420 18.74033 0.94088

familyhome1 -0.02965 0.02036 -1.45635 27.15914 0.14592

familyhome2 0.02977 0.02311 1.28816 43.27002 0.19828

cook1 -0.03411 0.02761 -1.23516 13.44125 0.21735

degree1 0.02951 0.02503 1.17904 38.73585 0.23894

gender_hisei1 0.00023 0.01287 0.01791 94.12385 0.98572

gender_hisei2 -0.00013 0.02374 -0.00559 400.48432 0.99555

gender_family1 0.01159 0.01779 0.65137 105.13481 0.51511

gender_family2 0.01243 0.02165 0.57408 91.99790 0.56617

gender_cook1 0.01675 0.01965 0.85237 330.20411 0.39442

gender_degree1 0.02921 0.02370 1.23244 67.21627 0.21836

HERBS SPICES GARLIC ONIONS

estimate std.error statistic df p.value

(Intercept) 0.52668 0.04796 10.98176 41.50345 0.00000

genderdummy 0.03314 0.04391 0.75460 122.79935 0.45080

hiseidummy1 -0.03648 0.02326 -1.56860 22.68165 0.11729

hiseidummy2 -0.03844 0.04236 -0.90752 39.87643 0.36451

familyhome1 0.04950 0.02786 1.77682 84.33414 0.07613.

familyhome2 0.05299 0.03244 1.63345 150.39097 0.10292

cook1 -0.00512 0.03616 -0.14148 29.81622 0.88754

degree1 0.01161 0.03570 0.32531 92.66170 0.74506

gender_hisei1 0.01169 0.01925 0.60712 161.68279 0.54401

gender_hisei2 0.01163 0.04591 0.25326 22.82080 0.80016

gender_family1 -0.00994 0.02948 -0.33727 44.44534 0.73603

gender_family2 0.03462 0.03072 1.12710 576.83458 0.26017

gender_cook1 -0.00363 0.03267 -0.11112 79.17526 0.91156

gender_degree1 -0.01873 0.03774 -0.49629 47.61279 0.61988

FRUITS

estimate std.error statistic df p.value

(Intercept) 3.00723 0.18618 16.15251 16.13213 0.00000

genderdummy -0.16407 0.14945 -1.09784 82.39950 0.27501

hiseidummy1 -0.03115 0.06578 -0.47356 96.35044 0.63689

hiseidummy2 -0.11212 0.14987 -0.74817 25.03470 0.45618

familyhome1 0.00144 0.10171 0.01413 32.05453 0.98875

familyhome2 0.04439 0.12679 0.35007 25.67660 0.72705

cook1 0.13911 0.15854 0.87741 9.72222 0.38245

degree1 0.09199 0.15820 0.58152 12.86967 0.56225

gender_hisei1 -0.01983 0.06777 -0.29264 64.61549 0.77043

gender_hisei2 0.00705 0.13929 0.05059 43.00752 0.95976

gender_family1 0.05199 0.12029 0.43224 13.74424 0.66653

gender_family2 -0.09818 0.11105 -0.88412 88.67906 0.37883

gender_cook1 -0.04530 0.11406 -0.39716 44.55426 0.69212

gender_degree1 0.08443 0.11905 0.70919 85.49412 0.47992

VEGETABLES

estimate std.error statistic df p.value

(Intercept) 2.30953 0.15277 15.11757 36.35971 0.00000

genderdummy -0.17314 0.13890 -1.24652 106.41534 0.21305

hiseidummy1 0.04468 0.06902 0.64739 32.30708 0.51762

hiseidummy2 0.17189 0.11951 1.43830 156.05812 0.15085

familyhome1 0.13782 0.08502 1.62115 129.64109 0.10549

familyhome2 0.15773 0.12080 1.30576 24.52515 0.19212

cook1 0.15012 0.13226 1.13506 13.92357 0.25679

degree1 0.17584 0.12708 1.38367 27.12559 0.16696

gender_hisei1 0.04706 0.05921 0.79488 247.03376 0.42699

gender_hisei2 0.07399 0.13467 0.54943 35.61416 0.58291

gender_family1 -0.00560 0.07965 -0.07029 620.35138 0.94398

gender_family2 0.03194 0.09780 0.32659 376.05289 0.74408

gender_cook1 0.02005 0.09920 0.20216 130.26330 0.83986

gender_degree1 -0.14610 0.12037 -1.21376 40.76639 0.22530

OLIVE OIL

estimate std.error statistic df p.value

(Intercept) 1.34859 0.06081 22.17844 30.30054 0.00000

genderdummy 0.02084 0.05348 0.38971 123.33878 0.69721

hiseidummy1 -0.00187 0.02426 -0.07727 90.52559 0.93849

hiseidummy2 0.08895 0.04842 1.83695 78.96882 0.06785.

familyhome1 -0.03578 0.03459 -1.03422 65.96886 0.30241

familyhome2 0.03872 0.04638 0.83488 26.35654 0.40488

cook1 0.01681 0.04753 0.35365 19.20779 0.72401

degree1 0.20562 0.04217 4.87597 156.37718 0.00000***

gender_hisei1 -0.01378 0.02549 -0.54037 49.90172 0.58960

gender_hisei2 0.03023 0.04608 0.65608 181.68732 0.51260

gender_family1 -0.00912 0.03639 -0.25058 39.53247 0.80242

gender_family2 -0.02055 0.04105 -0.50070 81.33201 0.61719

gender_cook1 -0.02712 0.04875 -0.55626 17.10761 0.57872

gender_degree1 0.05754 0.04290 1.34123 114.85075 0.18152

BREAD PASTA RICES

estimate std.error statistic df p.value

(Intercept) 2.34620 0.08158 28.75820 190.75716 0.00000

genderdummy 0.16398 0.08490 1.93151 93.59125 0.05453.

hiseidummy1 -0.02254 0.04318 -0.52191 26.05620 0.60219

hiseidummy2 -0.00734 0.08223 -0.08923 33.64656 0.92897

familyhome1 -0.08399 0.05830 -1.44071 32.03125 0.15090

familyhome2 0.05827 0.07090 0.82190 30.18798 0.41191

cook1 -0.15715 0.06352 -2.47402 59.84287 0.01402*

degree1 0.19845 0.07607 2.60899 29.50299 0.00962**

gender_hisei1 0.06542 0.03707 1.76454 126.52149 0.07884.

gender_hisei2 0.08027 0.08081 0.99328 38.66154 0.32152

gender_family1 0.02823 0.05129 0.55034 141.27809 0.58257

gender_family2 -0.08876 0.06388 -1.38945 84.24897 0.16591

gender_cook1 0.00783 0.06099 0.12837 102.61690 0.89796

gender_degree1 0.00128 0.06436 0.01989 253.69493 0.98415

POTATOES

estimate std.error statistic df p.value

(Intercept) 1.41505 0.11087 12.76346 18.62191 0.00000

genderdummy -0.01496 0.11340 -0.13192 16.82848 0.89523

hiseidummy1 0.03624 0.04803 0.75458 21.25419 0.45172

hiseidummy2 -0.01689 0.10261 -0.16464 14.99142 0.86946

familyhome1 -0.04515 0.05676 -0.79543 86.33537 0.42766

familyhome2 0.07658 0.07172 1.06777 49.63508 0.28739

cook1 0.02008 0.08090 0.24815 17.82219 0.80437

degree1 0.07660 0.10105 0.75811 11.48406 0.44961

gender_hisei1 -0.00366 0.04124 -0.08884 74.03482 0.92933

gender_hisei2 -0.01747 0.08935 -0.19553 30.79028 0.84525

gender_family1 0.00843 0.05497 0.15342 145.89892 0.87828

gender_family2 0.01957 0.07599 0.25749 30.54810 0.79716

gender_cook1 -0.10064 0.07117 -1.41405 39.35160 0.15948

gender_degree1 0.05797 0.10127 0.57243 11.41221 0.56791

RED MEAT AND PROCESSED MEAT

estimate std.error statistic df p.value

(Intercept) 1.41505 0.11087 12.76346 18.62191 0.00000

genderdummy -0.01496 0.11340 -0.13192 16.82848 0.89523

hiseidummy1 0.03624 0.04803 0.75458 21.25419 0.45172

hiseidummy2 -0.01689 0.10261 -0.16464 14.99142 0.86946

familyhome1 -0.04515 0.05676 -0.79543 86.33537 0.42766

familyhome2 0.07658 0.07172 1.06777 49.63508 0.28739

cook1 0.02008 0.08090 0.24815 17.82219 0.80437

degree1 0.07660 0.10105 0.75811 11.48406 0.44961

gender_hisei1 -0.00366 0.04124 -0.08884 74.03482 0.92933

gender_hisei2 -0.01747 0.08935 -0.19553 30.79028 0.84525

gender_family1 0.00843 0.05497 0.15342 145.89892 0.87828

gender_family2 0.01957 0.07599 0.25749 30.54810 0.79716

gender_cook1 -0.10064 0.07117 -1.41405 39.35160 0.15948

gender_degree1 0.05797 0.10127 0.57243 11.41221 0.56791

SWEETS

estimate std.error statistic df p.value

(Intercept) 6.14852 0.42055 14.62026 30.25729 0.00000

genderdummy 0.44365 0.44031 1.00759 22.70083 0.31458

hiseidummy1 -0.10333 0.18689 -0.55292 30.51138 0.58079

hiseidummy2 -0.07946 0.31351 -0.25347 263.11571 0.80011

familyhome1 -0.20453 0.23960 -0.85362 64.65279 0.39409

familyhome2 0.76729 0.31737 2.41762 28.20575 0.01630*

cook1 -0.28494 0.33071 -0.86158 18.64637 0.38970

degree1 -0.23186 0.41989 -0.55219 11.27319 0.58129

gender_hisei1 -0.12667 0.16116 -0.78597 183.44913 0.43259

gender_hisei2 0.03519 0.32161 0.10943 150.79572 0.91295

gender_family1 0.22625 0.24950 0.90681 42.55643 0.36534

gender_family2 0.41180 0.30451 1.35237 38.41152 0.17742

gender_cook1 0.13913 0.30153 0.46141 32.05643 0.64489

gender_degree1 0.13634 0.38106 0.35778 15.57003 0.72079

WHITE MEAT

estimate std.error statistic df p.value

(Intercept) 3.75729 0.23940 15.69451 22.95241 0.00000

genderdummy 0.20604 0.24815 0.83031 19.04905 0.40716

hiseidummy1 0.08290 0.10210 0.81194 29.91869 0.41761

hiseidummy2 -0.16790 0.20545 -0.81724 26.53339 0.41458

familyhome1 -0.25592 0.14196 -1.80276 30.14745 0.07264.

familyhome2 -0.18246 0.15975 -1.14216 54.85328 0.25449

cook1 0.13401 0.20835 0.64319 11.07217 0.52069

degree1 -0.08616 0.19290 -0.44664 21.86909 0.65552

gender_hisei1 0.00802 0.08827 0.09087 164.34376 0.92767

gender_hisei2 -0.12881 0.20054 -0.64234 31.23929 0.52125

gender_family1 0.04535 0.12058 0.37612 248.37754 0.70715

gender_family2 -0.02551 0.17066 -0.14948 30.86088 0.88130

gender_cook1 0.21763 0.17919 1.21454 19.13484 0.22570

gender_degree1 0.14162 0.19495 0.72643 20.67768 0.46826

FISH SEAFOOD

estimate std.error statistic df p.value

(Intercept) 5.63816 0.28414 19.84284 55.44421 0.00000

genderdummy -0.34731 0.30630 -1.13387 29.16552 0.25726

hiseidummy1 0.11945 0.15355 0.77797 15.66666 0.43686

hiseidummy2 -0.10780 0.29503 -0.36538 17.63881 0.71495

familyhome1 -0.11490 0.16027 -0.71688 251.69032 0.47370

familyhome2 0.05242 0.22914 0.22875 28.87165 0.81913

cook1 -0.30834 0.25533 -1.20761 14.33367 0.22763

degree1 -0.01816 0.23151 -0.07843 45.37225 0.93751

gender_hisei1 -0.11821 0.11109 -1.06414 662.56537 0.28765

gender_hisei2 -0.25198 0.22233 -1.13335 466.54474 0.25748

gender_family1 0.11745 0.16382 0.71694 156.05838 0.47366

gender_family2 0.04783 0.21034 0.22741 61.26122 0.82017

gender_cook1 0.06844 0.20721 0.33029 50.52206 0.74128

gender_degree1 0.01132 0.26183 0.04322 19.67790 0.96554

EGGS

estimate std.error statistic df p.value

(Intercept) 3.33188 0.22065 15.09995 26.09771 0.00000

genderdummy 0.87307 0.20633 4.23152 43.30087 0.00006***

hiseidummy1 0.02739 0.12944 0.21160 9.34658 0.83297

hiseidummy2 0.17536 0.20575 0.85232 18.93958 0.39663

familyhome1 -0.04071 0.14569 -0.27945 18.47171 0.78064

familyhome2 -0.13982 0.20017 -0.69852 11.48455 0.48692

cook1 0.37802 0.22045 1.71474 8.48247 0.09034.

degree1 0.14702 0.16543 0.88874 41.82970 0.37686

gender_hisei1 0.03102 0.11281 0.27499 13.66870 0.78405

gender_hisei2 0.19587 0.17990 1.08874 46.81874 0.27960

gender_family1 0.06809 0.12595 0.54061 49.68391 0.59031

gender_family2 -0.24701 0.14603 -1.69147 78.39034 0.09472.

gender_cook1 0.09618 0.17126 0.56160 17.87454 0.57599

gender_degree1 0.09019 0.18977 0.47526 17.65570 0.63592

LEGUMES

estimate std.error statistic df p.value

(Intercept) 3.55046 0.16323 21.75128 191.25550 0.00000

genderdummy 0.25302 0.17925 1.41156 48.87831 0.15971

hiseidummy1 0.00016 0.09888 0.00157 13.85429 0.99875

hiseidummy2 -0.11832 0.17010 -0.69555 26.69628 0.48755

familyhome1 -0.17415 0.11702 -1.48821 31.31488 0.13834

familyhome2 0.12056 0.13194 0.91374 56.93376 0.36201

cook1 -0.06445 0.16492 -0.39079 12.68465 0.69639

degree1 0.03285 0.13834 0.23743 71.77657 0.81257

gender_hisei1 0.11971 0.07746 1.54533 67.25068 0.12392

gender_hisei2 -0.09643 0.16230 -0.59416 37.49788 0.55311

gender_family1 -0.02795 0.11533 -0.24233 34.92775 0.80878

gender_family2 -0.00866 0.12699 -0.06817 92.56439 0.94572

gender_cook1 0.22859 0.12949 1.76535 49.15045 0.07910.

gender_degree1 -0.06268 0.13996 -0.44786 62.33926 0.65476

ALCOHOLIC DRINKS

estimate std.error statistic df p.value

(Intercept) 0.60196 0.09202 6.54167 62.19457 0.00000

genderdummy 0.07258 0.10158 0.71448 26.69350 0.47520

hiseidummy1 -0.05897 0.04195 -1.40574 48.02894 0.16031

hiseidummy2 0.08251 0.07877 1.04751 87.81081 0.29528

familyhome1 0.00878 0.06208 0.14147 29.11974 0.88755

familyhome2 0.03786 0.06122 0.61838 608.46148 0.53656

cook1 0.02225 0.07468 0.29791 24.04893 0.76587

degree1 -0.25129 0.06828 -3.68025 201.95055 0.00025**

gender_hisei1 -0.07851 0.04761 -1.64913 19.91644 0.09964

gender_hisei2 -0.03674 0.09102 -0.40370 23.79597 0.68658

gender_family1 0.02580 0.05501 0.46911 99.71333 0.63916

gender_family2 0.05418 0.08411 0.64415 15.84383 0.51972

gender_cook1 -0.03225 0.06875 -0.46910 44.15698 0.63917

gender_degree1 -0.08254 0.06694 -1.23305 326.84108 0.21803

FAST FOOD

estimate std.error statistic df p.value

(Intercept) 0.66301 0.02845 23.30781 30.56115 0.00000

genderdummy 0.07304 0.03174 2.30098 16.76068 0.02237*

hiseidummy1 0.02878 0.01322 2.17629 23.16141 0.03064*

hiseidummy2 0.01292 0.02672 0.48355 20.52444 0.62921

familyhome1 -0.02663 0.01731 -1.53811 34.79166 0.12552

familyhome2 0.01329 0.02586 0.51408 12.24127 0.60773

cook1 0.00127 0.02298 0.05523 16.63038 0.95601

degree1 -0.02042 0.02225 -0.91764 35.74257 0.35985

gender_hisei1 0.00926 0.01085 0.85414 211.34119 0.39399

gender_hisei2 -0.00500 0.02380 -0.20988 45.84748 0.83396

gender_family1 -0.02406 0.01652 -1.45663 53.06579 0.14670

gender_family2 -0.00089 0.01972 -0.04534 59.23243 0.96388

gender_cook1 -0.00041 0.02126 -0.01945 24.59427 0.98450

gender_degree1 0.00560 0.02111 0.26543 58.56965 0.79094

PRECOOKED FOOD

estimate std.error statistic df p.value

(Intercept) 1.02243 0.06142 16.64592 29.27544 0.00000

genderdummy -0.03637 0.06726 -0.54080 17.71214 0.58908

hiseidummy1 0.02788 0.02653 1.05094 36.48163 0.29419

hiseidummy2 0.01527 0.06850 0.22291 10.69833 0.82376

familyhome1 -0.02489 0.03639 -0.68412 41.32616 0.49446

familyhome2 0.00968 0.04874 0.19860 20.48342 0.84272

cook1 0.00734 0.05014 0.14650 15.55609 0.88363

degree1 -0.05662 0.04499 -1.25854 63.49299 0.20924

gender_hisei1 -0.00822 0.02300 -0.35749 280.50327 0.72099

gender_hisei2 -0.07997 0.04867 -1.64315 78.97637 0.10147

gender_family1 0.01436 0.04021 0.35711 21.01280 0.72127

gender_family2 -0.00006 0.04182 -0.00151 68.18746 0.99879

gender_cook1 0.00058 0.03992 0.01441 81.49954 0.98851

gender_degree1 0.02351 0.04604 0.51062 49.49138 0.61002

## Imputed data (m=30)

DAIRY PRODUCTS

estimate std.error statistic df p.value

(Intercept) 2.87813 0.13186 21.82731 259.9423 0.00000

genderdummy 0.15594 0.13995 1.11425 173.4054 0.26597

hiseidummy1 -0.07170 0.06285 -1.14081 164.1366 0.25476

hiseidummy2 -0.13345 0.11858 -1.12541 220.2641 0.26122

familyhome1 0.09747 0.08294 1.17530 231.5761 0.24071

familyhome2 0.08873 0.10535 0.84223 165.6309 0.40026

cook1 -0.03558 0.10589 -0.33601 135.0690 0.73708

degree1 0.02417 0.11418 0.21171 153.4778 0.83246

gender_hisei1 -0.05435 0.05991 -0.90721 222.8425 0.36495

gender_hisei2 -0.11649 0.11238 -1.03651 335.6297 0.30071

gender_family1 0.10105 0.08053 1.25475 290.2264 0.21044

gender_family2 0.04189 0.10434 0.40150 175.3906 0.68831

gender_cook1 0.00798 0.09759 0.08175 218.5290 0.93490

gender_degree1 -0.02651 0.11087 -0.23912 182.2589 0.81116

OLIVES NUTS SEEDS

estimate std.error statistic df p.value

(Intercept) 0.36516 0.03097 11.79265 237.6669 0.00000

genderdummy 0.05362 0.03149 1.70246 210.8685 0.08937.

hiseidummy1 -0.00650 0.01428 -0.45559 186.6190 0.64891

hiseidummy2 0.00300 0.02968 0.10109 140.0221 0.91953

familyhome1 -0.02931 0.01826 -1.60458 353.0391 0.10929

familyhome2 0.03180 0.02251 1.41259 292.8900 0.15848

cook1 -0.03676 0.02193 -1.67593 277.3187 0.09445.

degree1 0.02561 0.02826 0.90622 112.3514 0.36531

gender_hisei1 -0.00320 0.01419 -0.22525 194.1436 0.82188

gender_hisei2 0.00253 0.02688 0.09409 261.9901 0.92508

gender_family1 0.01080 0.01781 0.60626 445.6051 0.54465

gender_family2 0.01882 0.02204 0.85403 351.4439 0.39355

gender_cook1 0.01120 0.02161 0.51839 313.2409 0.60444

gender_degree1 0.02924 0.02550 1.14691 192.7765 0.25203

HERBS SPICES GARLIC ONIONS

estimate std.error statistic df p.value

(Intercept) 0.52976 0.04793 11.05184 199.9862 0.00000

genderdummy 0.04897 0.04859 1.00766 183.2770 0.31411

hiseidummy1 -0.03174 0.02150 -1.47607 189.6126 0.14056

hiseidummy2 -0.03260 0.04253 -0.76659 188.5670 0.44369

familyhome1 0.04463 0.02796 1.59630 313.1725 0.11106

familyhome2 0.04939 0.03711 1.33088 160.6185 0.18384

cook1 0.00805 0.03841 0.20954 114.9543 0.83411

degree1 0.00716 0.04047 0.17692 144.4253 0.85965

gender_hisei1 0.01526 0.02140 0.71334 195.6830 0.47597

gender_hisei2 0.01253 0.04004 0.31306 291.8241 0.75437

gender_family1 -0.01554 0.02660 -0.58401 495.5513 0.55948

gender_family2 0.02856 0.03530 0.80895 220.0516 0.41893

gender_cook1 -0.00114 0.03212 -0.03560 359.7201 0.97162

gender_degree1 0.00477 0.03874 0.12313 185.4542 0.90205

FRUITS

estimate std.error statistic df p.value

(Intercept) 2.91795 0.14944 19.52637 266.9874 0.00000

genderdummy -0.13166 0.15555 -0.84638 200.1565 0.39777

hiseidummy1 -0.05331 0.07188 -0.74174 158.9726 0.45862

hiseidummy2 -0.11583 0.12887 -0.89885 312.5683 0.36919

familyhome1 -0.02386 0.09361 -0.25493 244.6721 0.79889

familyhome2 0.08390 0.11910 0.70444 171.4656 0.48151

cook1 0.09464 0.11187 0.84597 206.9040 0.39800

degree1 0.06747 0.12552 0.53749 187.3594 0.59119

gender_hisei1 -0.01767 0.06393 -0.27632 371.8020 0.78242

gender_hisei2 -0.00933 0.12944 -0.07205 301.2707 0.94259

gender_family1 0.02474 0.08685 0.28480 467.4315 0.77592

gender_family2 -0.07751 0.10848 -0.71451 338.0908 0.47527

gender_cook1 -0.05571 0.10604 -0.52532 309.8601 0.59961

gender_degree1 0.10146 0.12722 0.79750 172.5510 0.42556

VEGETABLES

estimate std.error statistic df p.value

(Intercept) 2.22138 0.13860 16.02727 377.4459 0.00000

genderdummy -0.19600 0.14292 -1.37137 289.4243 0.17092

hiseidummy1 0.06578 0.06577 1.00015 224.0513 0.31775

hiseidummy2 0.14670 0.12709 1.15428 264.4940 0.24897

familyhome1 0.12250 0.08622 1.42074 362.2192 0.15606

familyhome2 0.16644 0.10530 1.58056 324.5446 0.11465

cook1 0.15832 0.11476 1.37957 144.8418 0.16838

degree1 0.09903 0.12269 0.80715 174.4348 0.41999

gender_hisei1 0.04720 0.06167 0.76538 377.1265 0.44443

gender_hisei2 0.04278 0.11910 0.35925 468.8988 0.71957

gender_family1 -0.00756 0.08634 -0.08758 357.9558 0.93025

gender_family2 0.05353 0.10665 0.50190 291.6714 0.61597

gender_cook1 0.01969 0.10290 0.19138 298.6960 0.84831

gender_degree1 -0.15974 0.11337 -1.40899 306.2285 0.15950

OLIVE OIL

estimate std.error statistic df p.value

(Intercept) 1.33985 0.06262 21.39529 142.03906 0.00000

genderdummy -0.00292 0.05878 -0.04962 206.42349 0.96044

hiseidummy1 0.01130 0.02924 0.38643 113.16241 0.69937

hiseidummy2 0.08689 0.05335 1.62862 169.08744 0.10411

familyhome1 -0.04446 0.03468 -1.28201 296.07330 0.20051

familyhome2 0.03590 0.04425 0.81117 195.99419 0.41771

cook1 0.02088 0.04175 0.50016 233.25319 0.61721

degree1 0.21743 0.05428 4.00590 98.76154 0.00007***

gender_hisei1 -0.01966 0.02555 -0.76949 243.26097 0.44202

gender_hisei2 0.03498 0.04771 0.73316 390.16702 0.46385

gender_family1 -0.00768 0.03317 -0.23152 440.80357 0.81702

gender_family2 -0.01542 0.04211 -0.36617 280.53922 0.71441

gender_cook1 -0.01878 0.04057 -0.46280 290.46049 0.64374

gender_degree1 0.04034 0.05062 0.79696 133.75147 0.42590

BREAD PASTA RICES

estimate std.error statistic df p.value

(Intercept) 2.33068 0.09422 24.73616 185.2513 0.00000

genderdummy 0.16500 0.09093 1.81461 235.5861 0.07024.

hiseidummy1 -0.00576 0.04363 -0.13212 147.1271 0.89495

hiseidummy2 -0.03175 0.08491 -0.37388 160.0205 0.70867

familyhome1 -0.06702 0.05527 -1.21264 271.2892 0.22590

familyhome2 0.07254 0.06707 1.08155 258.9044 0.28003

cook1 -0.12587 0.07368 -1.70830 121.5201 0.08826.

degree1 0.20504 0.07301 2.80855 226.8911 0.00519**

gender_hisei1 0.07581 0.03962 1.91315 275.9014 0.05636.

gender_hisei2 0.05703 0.07675 0.74302 325.8551 0.45785

gender_family1 0.00679 0.05213 0.13026 454.8968 0.89642

gender_family2 -0.07122 0.06600 -1.07904 294.1296 0.28114

gender_cook1 0.01551 0.06726 0.23064 200.7093 0.81770

gender_degree1 0.02442 0.07229 0.33780 243.7566 0.73567

POTATOES

estimate std.error statistic df p.value

(Intercept) 2.33068 0.09422 24.73616 185.2513 0.00000

genderdummy 0.16500 0.09093 1.81461 235.5861 0.07024.

hiseidummy1 -0.00576 0.04363 -0.13212 147.1271 0.89495

hiseidummy2 -0.03175 0.08491 -0.37388 160.0205 0.70867

familyhome1 -0.06702 0.05527 -1.21264 271.2892 0.22590

familyhome2 0.07254 0.06707 1.08155 258.9044 0.28003

cook1 -0.12587 0.07368 -1.70830 121.5201 0.08826.

degree1 0.20504 0.07301 2.80855 226.8911 0.00519**

gender_hisei1 0.07581 0.03962 1.91315 275.9014 0.05636.

gender_hisei2 0.05703 0.07675 0.74302 325.8551 0.45785

gender_family1 0.00679 0.05213 0.13026 454.8968 0.89642

gender_family2 -0.07122 0.06600 -1.07904 294.1296 0.28114

gender_cook1 0.01551 0.06726 0.23064 200.7093 0.81770

gender_degree1 0.02442 0.07229 0.33780 243.7566 0.73567

RED MEAT AND PROCESSED MEAT

estimate std.error statistic df p.value

(Intercept) 13.45212 0.64650 20.80758 135.7524 0.00000

genderdummy 1.01169 0.59095 1.71197 233.3450 0.08780.

hiseidummy1 0.56208 0.30531 1.84099 103.9807 0.06648.

hiseidummy2 -0.47788 0.52310 -0.91354 222.4089 0.36159

familyhome1 -0.30948 0.37260 -0.83059 205.9239 0.40678

familyhome2 -0.45621 0.49429 -0.92295 121.4519 0.35667

cook1 -0.21888 0.44957 -0.48687 167.2196 0.62666

degree1 -0.00664 0.50345 -0.01320 155.0737 0.98948

gender_hisei1 0.17756 0.25024 0.70955 347.2764 0.47846

gender_hisei2 0.04235 0.51693 0.08193 242.2966 0.93475

gender_family1 0.28302 0.36823 0.76859 223.3239 0.44266

gender_family2 0.55548 0.42635 1.30286 305.8750 0.19349

gender_cook1 0.25469 0.43564 0.58463 203.4716 0.55917

gender_degree1 0.44031 0.47224 0.93238 232.4853 0.35179

SWEETS

estimate std.error statistic df p.value

(Intercept) 6.02206 0.41626 14.46690 181.6742 0.00000

genderdummy 0.39651 0.40500 0.97904 217.4404 0.32833

hiseidummy1 -0.08058 0.17615 -0.45746 256.4867 0.64766

hiseidummy2 0.04287 0.34595 0.12393 269.1776 0.90145

familyhome1 -0.24125 0.24959 -0.96657 225.0122 0.33452

familyhome2 0.52987 0.29327 1.80677 273.7264 0.07178.

cook1 -0.51294 0.30657 -1.67313 162.4788 0.09532.

degree1 -0.25472 0.36383 -0.70010 113.9190 0.48439

gender_hisei1 -0.09672 0.18553 -0.52131 179.6905 0.60252

gender_hisei2 0.12838 0.35687 0.35973 214.1995 0.71930

gender_family1 0.20649 0.23976 0.86124 306.8237 0.38978

gender_family2 0.19879 0.29257 0.67946 278.9699 0.49736

gender_cook1 0.04218 0.29765 0.14171 194.4543 0.88740

gender_degree1 0.08375 0.33025 0.25360 189.4671 0.79997

WHITE MEAT

estimate std.error statistic df p.value

(Intercept) 3.72291 0.22260 16.72486 213.7003 0.00000

genderdummy 0.23011 0.21105 1.09034 322.0657 0.27631

hiseidummy1 0.03645 0.10543 0.34567 146.4696 0.72980

hiseidummy2 -0.26333 0.20893 -1.26034 144.3657 0.20839

familyhome1 -0.25456 0.14109 -1.80423 183.6216 0.07205.

familyhome2 -0.19487 0.17122 -1.13811 176.8268 0.25585

cook1 0.15751 0.16995 0.92685 152.7578 0.35464

degree1 -0.13561 0.17584 -0.77121 230.9539 0.44110

gender_hisei1 0.02795 0.09896 0.28239 214.8197 0.77781

gender_hisei2 -0.12998 0.19080 -0.68126 257.0235 0.49616

gender_family1 0.00018 0.13669 0.00133 226.7667 0.99894

gender_family2 0.02262 0.16078 0.14067 273.8040 0.88821

gender_cook1 0.18674 0.15110 1.23592 349.8959 0.21732

gender_degree1 0.12471 0.17827 0.69959 209.8339 0.48465

FISH SEAFOOD

estimate std.error statistic df p.value

(Intercept) 5.63502 0.30849 18.26619 151.8805 0.00000

genderdummy -0.17559 0.29902 -0.58724 182.0152 0.55747

hiseidummy1 0.09872 0.14602 0.67607 112.6249 0.49950

hiseidummy2 -0.08004 0.26383 -0.30337 178.1703 0.76181

familyhome1 -0.08943 0.18100 -0.49408 211.1154 0.62160

familyhome2 0.02578 0.22014 0.11712 199.6253 0.90684

cook1 -0.25885 0.21972 -1.17805 164.8956 0.23967

degree1 -0.00952 0.22714 -0.04191 257.2367 0.96659

gender_hisei1 -0.08133 0.12802 -0.63527 235.6727 0.52572

gender_hisei2 -0.22587 0.25207 -0.89607 242.0838 0.37091

gender_family1 -0.01409 0.17192 -0.08195 312.3134 0.93474

gender_family2 0.08397 0.22458 0.37391 176.0266 0.70873

gender_cook1 -0.02164 0.20565 -0.10523 256.2244 0.91626

gender_degree1 0.09629 0.23891 0.40306 181.6449 0.68718

EGGS

estimate std.error statistic df p.value

(Intercept) 3.23501 0.22742 14.22458 132.5429 0.00000

genderdummy 0.77745 0.21470 3.62110 182.0224 0.00035**

hiseidummy1 0.02120 0.09412 0.22526 199.9819 0.82196

hiseidummy2 0.16812 0.19781 0.84991 139.9260 0.39615

familyhome1 -0.08498 0.12917 -0.65786 220.1243 0.51121

familyhome2 -0.08401 0.15975 -0.52590 186.5022 0.59940

cook1 0.35711 0.16280 2.19346 139.0565 0.02915*

degree1 0.05369 0.17864 0.30054 144.2344 0.76401

gender_hisei1 0.01652 0.09117 0.18118 250.3796 0.85636

gender_hisei2 0.18718 0.19157 0.97709 166.6711 0.32942

gender_family1 0.07161 0.13343 0.53666 178.1308 0.59196

gender_family2 -0.18366 0.15212 -1.20733 262.4717 0.22839

gender_cook1 0.08369 0.15663 0.53435 172.0412 0.59355

gender_degree1 0.05158 0.17129 0.30111 183.2831 0.76357

LEGUMES

estimate std.error statistic df p.value

(Intercept) 3.51760 0.17370 20.25070 294.0138 0.00000

genderdummy 0.32889 0.18005 1.82662 223.6070 0.06865.

hiseidummy1 0.01562 0.08682 0.17994 138.0860 0.85731

hiseidummy2 -0.18815 0.16049 -1.17234 203.5995 0.24190

familyhome1 -0.17015 0.10600 -1.60512 332.9609 0.10941

familyhome2 0.09414 0.13934 0.67560 177.2762 0.49976

cook1 -0.12821 0.12923 -0.99212 235.1132 0.32186

degree1 0.04576 0.15505 0.29515 141.8493 0.76806

gender_hisei1 0.11864 0.07927 1.49655 241.1597 0.13546

gender_hisei2 -0.09714 0.15364 -0.63223 280.1422 0.52767

gender_family1 -0.08915 0.10778 -0.82713 289.3940 0.40875

gender_family2 -0.00167 0.14733 -0.01133 130.9684 0.99097

gender_cook1 0.20017 0.13200 1.51643 202.9012 0.13036

gender_degree1 -0.05102 0.14602 -0.34939 201.5225 0.72702

ALCOHOLIC DRINKS

estimate std.error statistic df p.value

(Intercept) 0.59882 0.09371 6.39027 246.32450 0.00000

genderdummy 0.10999 0.10037 1.09585 157.88004 0.27422

hiseidummy1 -0.07651 0.05330 -1.43537 76.49963 0.15245

hiseidummy2 0.05662 0.08382 0.67553 217.64595 0.49997

familyhome1 0.01210 0.06451 0.18757 130.15869 0.85137

familyhome2 0.03524 0.07614 0.46286 145.10484 0.64387

cook1 -0.00229 0.07446 -0.03071 137.47780 0.97553

degree1 -0.25204 0.07561 -3.33339 228.65987 0.00099***

gender_hisei1 -0.07931 0.04828 -1.64271 108.34268 0.10172

gender_hisei2 -0.02560 0.08917 -0.28709 149.11517 0.77429

gender_family1 0.01562 0.06018 0.25952 191.64646 0.79545

gender_family2 0.05121 0.07713 0.66395 135.83212 0.50734

gender_cook1 -0.03767 0.07246 -0.51978 158.83249 0.60369

gender_degree1 -0.07676 0.07497 -1.02397 243.37567 0.30685

FAST FOOD

estimate std.error statistic df p.value

(Intercept) 0.66910 0.02993 22.35294 134.5271 0.00000

genderdummy 0.07241 0.02717 2.66548 242.7461 0.00791**

hiseidummy1 0.02867 0.01380 2.07791 114.1461 0.03818*

hiseidummy2 0.00954 0.02492 0.38299 182.2076 0.70188

familyhome1 -0.02590 0.01771 -1.46219 172.3625 0.14426

familyhome2 0.00763 0.02177 0.35041 154.6259 0.72616

cook1 0.00203 0.02225 0.09106 117.9081 0.92748

degree1 -0.00932 0.02327 -0.40053 154.9866 0.68892

gender_hisei1 0.00827 0.01125 0.73490 448.2984 0.46271

gender_hisei2 -0.00394 0.02176 -0.18106 553.7428 0.85638

gender_family1 -0.02424 0.01635 -1.48208 303.2666 0.13889

gender_family2 -0.00336 0.01979 -0.17001 295.0860 0.86507

gender_cook1 -0.00254 0.01962 -0.12942 243.6120 0.89708

gender_degree1 0.00317 0.02181 0.14549 233.3767 0.88437

PRECOOKED FOOD

estimate std.error statistic df p.value

(Intercept) 1.01265 0.05676 17.84012 214.0028 0.00000

genderdummy -0.04189 0.05378 -0.77895 324.6795 0.43640

hiseidummy1 0.03907 0.02597 1.50429 178.4285 0.13318

hiseidummy2 0.02401 0.05072 0.47341 192.3768 0.63614

familyhome1 -0.01528 0.03208 -0.47635 457.9582 0.63405

familyhome2 0.01586 0.04586 0.34572 135.1084 0.72971

cook1 0.01639 0.04474 0.36635 129.8584 0.71427

degree1 -0.07890 0.04519 -1.74599 218.9243 0.08147.

gender_hisei1 -0.01338 0.02630 -0.50863 165.4727 0.61125

gender_hisei2 -0.06797 0.05614 -1.21082 112.7871 0.22658

gender_family1 0.01712 0.03201 0.53472 467.0112 0.59310

gender_family2 -0.00229 0.03905 -0.05858 419.7205 0.95331

gender_cook1 -0.00345 0.04176 -0.08259 190.4508 0.93421

gender_degree1 0.01794 0.04444 0.40377 246.8666 0.68657

# Supplementary tables and figures

## Supplementary tables

Table A. Foods and food groups collected in questionnaire.

| Food group | Foods |
| --- | --- |
| Dairy products | Whole milk, semi-skimmed milk, skimmed milk, yogurt, curd, Petit Suise, Actimel, fresh cheese, semi-cured cheese, cheese in portions, mozzarella cheese, and sliced cheese. |
| Eggs, meat and fish | Eggs, chicken or turkey with skin, chicken or turkey without skin, veal, pork meat, mutton, rabbit meat, hamburger, meatball, bacon, sausage, blood sausage, cooked ham, Serrano ham, mortadella, other sausages, sobrassada, pate, fried fish, grilled fish, salted fish, smoked fish, canned fish, octopus/squid, seafood, and clams. |
| Vegetables | Swiss chard or spinach, cauliflower, green beans, asparagus, artichokes, aubergines, mushrooms, lettuce or endive, tomato, onion, carrots, leek, red pepper, canned vegetables, and baked potato. |
| Legumes | Lentils, chickpeas, beans, peas, others. |
| Cereals | Rice, noodle, spaghetti or macaroni, white bread, toasted bread, sliced bread, others. |
| Oils and fats | Olive oil, sunflower oil, butter, margarine, mayonnaise, and olives. |
| Fruit | Orange, mandarin, banana, apple or pear, avocado, strawberry or cherry, peach, fresh figs, kiwi, watermelon or melon, grapes, pineapple, fruit in syrup, nuts, figs, orange juice, bottled fruit juices. |
| Sweets and desserts | Jam, churros, María cookies, chocolate cookies, croissant, ensaimada, Donuts, cupcake or cake, pies, brownie, flan, custard, ice cream, chocolate mousse, chocolate or chocolate box, sugary breakfast cereals. |
| Beverages | Coffee, tea, soda, and sugary soft drinks, wine, beer, spirits, and distilled drinks. |
| Spices | Salt, sugar, garlic, spicy spices, ketchup, and mustard. |
| Precooked products | Croquettes, pasties, breaded squids, surimi sticks, potato omelette, paella, packed salad, soup or cream, cannelloni, Chinese noodle, and Andalusian gazpacho. |
| Fast food | Pizza, hamburger (McDonald's), Kebab, mixed sandwich, sandwich, hotdog, fried chicken wings, chips, snacks, and bag of chips. |

Table B. Food groups in the Mediterranean diet pyramid and foods from the FFQ

| Food group | Servings | Foods |
| --- | --- | --- |
| *Daily* |  |  |
| Dairy products | 2 | Whole milk, semi-skimmed milk, skimmed milk, yogurt, curd, Petit Suise, Actimel, fresh cheese, semi-cured cheese, cured cheese, cheese in portions, mozzarella cheese, and sliced cheese. |
| Olives, nuts, seeds | 1-2 | Olives, nuts. |
| Herbs, spices, garlic, onions | - | Onion and garlic. |
| Fruits | 3-6 | Orange, mandarin, banana, apple or pear, avocado, strawberry or cherry, peach, fresh figs, kiwi, watermelon or melon, grapes, pineapple, figs, orange juice, bottled fruit juices. |
| Vegetables | ≥ 6 | Swiss chard or spinach, cauliflower, green beans, asparagus, artichokes, aubergines, mushrooms, lettuce or endive, tomato, carrots, leek, red pepper, and canned vegetables. |
| Olive oil | 3 | Olive oil. |
| Bread, pasta, rice, other cereals | 3-6 | Rice, noodle, spaghetti or macaroni, white bread, toasted bread, sliced bread and others. |
| *Weekly* |  |  |
| Potatoes | ≤ 3 | Baked potato. |
| Red meat and processed meat | < 2 | Veal, pork meat, mutton, hamburger, meatball, bacon, sausage, blood sausage, cooked ham, Serrano ham, mortadella, other sausages, sobrassada, and pate. |
| Sweets | ≤ 2 | Fruit in syrup, jam, cupcake or cake, brownie, flan, custard, ice cream, chocolate mousse, chocolate or chocolate box, and sugary soft drinks. |
| White meat | 2 | Chicken or turkey with skin, chicken or turkey without skin, and rabbit meat. |
| Fish, seafood | ≥ 2 | Fried fish, grilled fish, salted fish, smoked fish, canned fish, octopus/squid, seafood, and clams. |
| Eggs | 2-4 | Eggs. |
| Legumes | ≥ 2 | Lentils, chickpeas, beans, peas, others. |
| *Other food groups of interest* |  |  |
| Alcoholic drinks (daily) | 1-2 AU/d | Wine, beer, liquors, spirits. |
| Fast food (weekly) | 0 | Pizza, hamburger (McDonald's), Kebab, mixed sandwich, sandwich, hotdog, fried chicken wings, chips, snacks, and bag of chips. |
| Precooked food (weekly) | 0 | Croquettes, pasties, breaded squids, surimi sticks, potato omelette, paella, packed salad, soup or cream, cannelloni, Chinese noodle, and Andalusian gazpacho. |

Abbreviations: AU: Alcohol Units

Table C. Occupations collected in the questionnaire: ISCO, ISEI-08 and SES

|  | Occupations collected in the questionnaire | ISCO-08 | ISEI-08 | SES Categories |
| --- | --- | --- | --- | --- |
| 1 | Management in companies and the public administration | 1 | 62 | High |
| 2 | Scientific and cultural technicians and professionals | 2 | 65 | High |
| 3 | Clerical workers | 4 | 41 | Medium |
| 4 | Support technicians and professionals | 3 | 51 | Medium |
| 5 | Hospitality and retail workers | 5 | 31 | Low |
| 6 | Armed forces | 0 | 53 | Medium |
| 7 | Mining, construction and manufacturing workers | 7-8 | 34 | Low |
| 8 | Agricultural and fishing workers | 6 | 18 | Low |
| 9 | Unskilled workers | 9 | 20 | Low |
| 10 | Non-working | - | 10 | Low |
| 11 | Retired | - | 10 | Low |
| 12 | Unemployed | - | 10 | Low |
| 13 | Civil servant | 2-3-4 | 52 | Medium |

Abbreviations: ISCO: International Standard Classification of Occupations; ISEI-08: International Socio-Economic Index of occupational status; SES: socioeconomic status.

Table D. Variables sorted by percentage of missing

| Variable | Percentage |
| --- | --- |
| Dairy products | 35.82% |
| Olives, nuts, seeds | 35.82% |
| Herbs, spices, garlic, onions | 35.82% |
| Fruits | 35.82% |
| Vegetables | 35.82% |
| Olive oil | 35.82% |
| Bread, pasta, rice, other cereals | 35.82% |
| Potatoes | 35.82% |
| Red meat and processed meat | 35.82% |
| Sweets | 35.82% |
| White meat | 35.82% |
| Fish, seafood | 35.82% |
| Eggs | 35.82% |
| Legumes | 35.82% |
| Alcoholic drinks (daily) | 35.82% |
| Fast food (weekly) | 35.82% |
| Precooked food (weekly) | 35.82% |
|  |  |

Table E. Independent variables: dummy coding

| Group | Number | Gender | SES (1) | SES (2) | Family home (1) | Family home (2) | | Cooking | Degree |
| --- | --- | --- | --- | --- | --- | --- | --- | --- | --- |
| Men-ISEI-08>62-Urban-Cook-Health | 1 | 1 | 1 | 1 | 1 | 1 | 1 | | 1 |
| Men-ISEI-08>62-Urban-nocook-Health | 2 | 1 | 1 | 1 | 1 | 1 | -1 | | 1 |
| Men-ISEI-08>62-Suburban-Cook-Health | 3 | 1 | 1 | 1 | 1 | -1 | 1 | | 1 |
| Men-ISEI-08>62-Suburban-nocook-Health | 4 | 1 | 1 | 1 | 1 | -1 | -1 | | 1 |
| Men-ISEI-08>62-Rural-Cook-Health | 5 | 1 | 1 | 1 | -2 | 0 | 1 | | 1 |
| Men-ISEI-08>62-Rural-nocook-Health | 6 | 1 | 1 | 1 | -2 | 0 | -1 | | 1 |
| Men-36<ISEI-08<62-Urban-Cook-Health | 7 | 1 | 1 | -1 | 1 | 1 | 1 | | 1 |
| Men-36<ISEI-08<62-Urban-nocook-Health | 8 | 1 | 1 | -1 | 1 | 1 | -1 | | 1 |
| Men-36<ISEI-08<62-Suburban-Cook-Health | 9 | 1 | 1 | -1 | 1 | -1 | 1 | | 1 |
| Men-36<ISEI-08<62-Suburban-nocook-Health | 10 | 1 | 1 | -1 | 1 | -1 | -1 | | 1 |
| Men-36<ISEI-08<62-Rural-Cook-Health | 11 | 1 | 1 | -1 | -2 | 0 | 1 | | 1 |
| Men-36<ISEI-08<62-Rural-nocook-Health | 12 | 1 | 1 | -1 | -2 | 0 | -1 | | 1 |
| Men-ISEI-08<36-Urban-Cook-Health | 13 | 1 | -2 | 0 | 1 | 1 | 1 | | 1 |
| Men-ISEI-08<36-Urban-nocook-Health | 14 | 1 | -2 | 0 | 1 | 1 | -1 | | 1 |
| Men-ISEI-08<36-Suburban-Cook-Health | 15 | 1 | -2 | 0 | 1 | -1 | 1 | | 1 |
| Men-ISEI-08<36-Suburban-nocook-Health | 16 | 1 | -2 | 0 | 1 | -1 | -1 | | 1 |
| Men-ISEI-08<36-Rural-Cook-Health | 17 | 1 | -2 | 0 | -2 | 0 | 1 | | 1 |
| Men-ISEI-08<36-Rural-nocook-Health | 18 | 1 | -2 | 0 | -2 | 0 | -1 | | 1 |
| Women-ISEI-08>62-Urban-Cook-Health | 19 | -1 | 1 | 1 | 1 | 1 | 1 | | 1 |
| Women-ISEI-08>62-Urban-nocook-Health | 20 | -1 | 1 | 1 | 1 | 1 | -1 | | 1 |
| Women-ISEI-08>62-Suburban-Cook-Health | 21 | -1 | 1 | 1 | 1 | -1 | 1 | | 1 |
| Women-ISEI-08>62-Suburban-nocook-Health | 22 | -1 | 1 | 1 | 1 | -1 | -1 | | 1 |
| Women-ISEI-08>62-Rural-Cook-Health | 23 | -1 | 1 | 1 | -2 | 0 | 1 | | 1 |
| Women-ISEI-08>62-Rural-nocook-Health | 24 | -1 | 1 | 1 | -2 | 0 | -1 | | 1 |
| Women-36<ISEI-08<62-Urban-Cook-Health | 25 | -1 | 1 | -1 | 1 | 1 | 1 | | 1 |
| Women-36<ISEI-08<62-Urban-nocook-Health | 26 | -1 | 1 | -1 | 1 | 1 | -1 | | 1 |
| Women-36<ISEI-08<62-Suburban-Cook-Health | 27 | -1 | 1 | -1 | 1 | -1 | 1 | | 1 |
| Women-36<ISEI-08<62-Suburban-nocook-Health | 28 | -1 | 1 | -1 | 1 | -1 | -1 | | 1 |
| Women-36<ISEI-08<62-Rural-Cook-Health | 29 | -1 | 1 | -1 | -2 | 0 | 1 | | 1 |
| Women-36<ISEI-08<62-Rural-nocook-Health | 30 | -1 | 1 | -1 | -2 | 0 | -1 | | 1 |
| Women-ISEI-08<36-Urban-Cook-Health | 31 | -1 | -2 | 0 | 1 | 1 | 1 | | 1 |
| Women-ISEI-08<36-Urban-nocook-Health | 32 | -1 | -2 | 0 | 1 | 1 | -1 | | 1 |
| Women-ISEI-08<36-Suburban-Cook-Health | 33 | -1 | -2 | 0 | 1 | -1 | 1 | | 1 |
| Women-ISEI-08<36-Suburban-nocook-Health | 34 | -1 | -2 | 0 | 1 | -1 | -1 | | 1 |
| Women-ISEI-08<36-Rural-Cook-Health | 35 | -1 | -2 | 0 | -2 | 0 | 1 | | 1 |
| Women-ISEI-08<36-Rural-nocook-Health | 36 | -1 | -2 | 0 | -2 | 0 | -1 | | 1 |
| Men-ISEI-08>62-Urban-Cook-Social | 37 | 1 | 1 | 1 | 1 | 1 | 1 | | -1 |
| Men-ISEI-08>62-Urban-nocook-Social | 38 | 1 | 1 | 1 | 1 | 1 | -1 | | -1 |
| Men-ISEI-08>62-Suburban-Cook-Social | 39 | 1 | 1 | 1 | 1 | -1 | 1 | | -1 |
| Men-ISEI-08>62-Suburban-nocook-Social | 40 | 1 | 1 | 1 | 1 | -1 | -1 | | -1 |
| Men-ISEI-08>62-Rural-Cook-Social | 41 | 1 | 1 | 1 | -2 | 0 | 1 | | -1 |
| Men-ISEI-08>62-Rural-nocook-Social | 42 | 1 | 1 | 1 | -2 | 0 | -1 | | -1 |
| Men-36<ISEI-08<62-Urban-Cook-Social | 43 | 1 | 1 | -1 | 1 | 1 | 1 | | -1 |
| Men-36<ISEI-08<62-Urban-nocook-Social | 44 | 1 | 1 | -1 | 1 | 1 | -1 | | -1 |
| Men-36<ISEI-08<62-Suburban-Cook-Social | 45 | 1 | 1 | -1 | 1 | -1 | 1 | | -1 |
| Men-36<ISEI-08<62-Suburban-nocook-Social | 46 | 1 | 1 | -1 | 1 | -1 | -1 | | -1 |
| Men-36<ISEI-08<62-Rural-Cook-Social | 47 | 1 | 1 | -1 | -2 | 0 | 1 | | -1 |
| Men-36<ISEI-08<62-Rural-nocook-Social | 48 | 1 | 1 | -1 | -2 | 0 | -1 | | -1 |
| Men-ISEI-08<36-Urban-Cook-Social | 49 | 1 | -2 | 0 | 1 | 1 | 1 | | -1 |
| Men-ISEI-08<36-Urban-nocook-Social | 50 | 1 | -2 | 0 | 1 | 1 | -1 | | -1 |
| Men-ISEI-08<36-Suburban-Cook-Social | 51 | 1 | -2 | 0 | 1 | -1 | 1 | | -1 |
| Men-ISEI-08<36-Suburban-nocook-Social | 52 | 1 | -2 | 0 | 1 | -1 | -1 | | -1 |
| Men-ISEI-08<36-Rural-Cook-Social | 53 | 1 | -2 | 0 | -2 | 0 | 1 | | -1 |
| Men-ISEI-08<36-Rural-nocook-Social | 54 | 1 | -2 | 0 | -2 | 0 | -1 | | -1 |
| Women-ISEI-08>62-Urban-Cook-Social | 55 | -1 | 1 | 1 | 1 | 1 | 1 | | -1 |
| Women-ISEI-08>62-Urban-nocook-Social | 56 | -1 | 1 | 1 | 1 | 1 | -1 | | -1 |
| Women-ISEI-08>62-Suburban-Cook-Social | 57 | -1 | 1 | 1 | 1 | -1 | 1 | | -1 |
| Women-ISEI-08>62-Suburban-nocook-Social | 58 | -1 | 1 | 1 | 1 | -1 | -1 | | -1 |
| Women-ISEI-08>62-Rural-Cook-Social | 59 | -1 | 1 | 1 | -2 | 0 | 1 | | -1 |
| Women-ISEI-08>62-Rural-nocook-Social | 60 | -1 | 1 | 1 | -2 | 0 | -1 | | -1 |
| Women-36<ISEI-08<62-Urban-Cook-Social | 61 | -1 | 1 | -1 | 1 | 1 | 1 | | -1 |
| Women-36<ISEI-08<62-Urban-nocook-Social | 62 | -1 | 1 | -1 | 1 | 1 | -1 | | -1 |
| Women-36<ISEI-08<62-Suburban-Cook-Social | 63 | -1 | 1 | -1 | 1 | -1 | 1 | | -1 |
| Women-36<ISEI-08<62-Suburban-nocook-Social | 64 | -1 | 1 | -1 | 1 | -1 | -1 | | -1 |
| Women-36<ISEI-08<62-Rural-Cook-Social | 65 | -1 | 1 | -1 | -2 | 0 | 1 | | -1 |
| Women-36<ISEI-08<62-Rural-nocook-Social | 66 | -1 | 1 | -1 | -2 | 0 | -1 | | -1 |
| Women-ISEI-08<36-Urban-Cook-Social | 67 | -1 | -2 | 0 | 1 | 1 | 1 | | -1 |
| Women-ISEI-08<36-Urban-nocook-Social | 68 | -1 | -2 | 0 | 1 | 1 | -1 | | -1 |
| Women-ISEI-08<36-Suburban-Cook-Social | 69 | -1 | -2 | 0 | 1 | -1 | 1 | | -1 |
| Women-ISEI-08<36-Suburban-nocook-Social | 70 | -1 | -2 | 0 | 1 | -1 | -1 | | -1 |
| Women-ISEI-08<36-Rural-Cook-Social | 71 | -1 | -2 | 0 | -2 | 0 | 1 | | -1 |
| Women-ISEI-08<36-Rural-nocook-Social | 72 | -1 | -2 | 0 | -2 | 0 | -1 | | -1 |

Table F. Summary of interactions between gender and the other social determinants across food groups from fitted models

| Food group | Men | | | | | | Women | | | | | |
| --- | --- | --- | --- | --- | --- | --- | --- | --- | --- | --- | --- | --- |
| *Daily* | SES (1) | SES (2) | Family home (1) | Family home (2) | CFHS | Degree | SES (1) | SES (2) | Family home (1) | Family home (2) | CFHS | Degree |
| Dairy |  |  |  |  |  |  |  |  |  |  |  |  |
| Olives, nuts, seeds |  |  |  |  |  |  |  |  |  |  |  |  |
| Herbs, spices, garlic, onions |  |  |  |  |  |  |  |  |  |  |  |  |
| Fruits |  |  |  |  |  |  |  |  |  |  |  |  |
| Vegetables |  |  |  |  |  |  |  |  |  |  |  |  |
| Olive oil |  |  |  |  |  |  |  |  |  |  |  |  |
| Bread, pasta, rice, other cereals |  |  |  |  |  |  |  |  |  |  |  |  |
| *Weekly* |  |  |  |  |  |  |  |  |  |  |  |  |
| Potatoes |  |  |  |  |  |  |  |  |  |  |  |  |
| Red meat and processed meat |  |  |  |  |  |  |  |  |  |  |  |  |
| Sweets |  |  |  |  |  |  |  |  |  |  |  |  |
| White meat |  |  |  |  |  |  |  |  |  |  |  |  |
| Fish, seafood |  |  |  |  |  |  |  |  |  |  |  |  |
| Eggs |  |  |  |  |  |  |  |  |  |  |  |  |
| Legumes |  |  |  |  |  |  |  |  |  |  |  |  |
| *Daily consumption of other food groups of interest* | | | |  |  |  |  |  |  |  |  |  |
| Alcoholic drinks |  |  |  |  |  |  |  |  |  |  |  |  |
| Fast food |  |  |  |  |  |  |  |  |  |  |  |  |
| Precooked food |  |  |  |  |  |  |  |  |  |  |  |  |

Abbreviations: AU: Alcohol Units; CFHS: Cooks for him or herself

Table G. Results from complete-case and imputed data regressions

| Food group | Gender | Socioeconomic position | | Family home | | CFHS | Degree | Interactions | | | | |  |
| --- | --- | --- | --- | --- | --- | --- | --- | --- | --- | --- | --- | --- | --- |
| *Daily* |  | SES (1) | SES (2) | Family home (1) | Family home (2) | CFHS | Degree | Gender x SES (1) | Gender x SES (2) | Gender x Family home (1) | Gender x Family home (2) | Gender x CFHS | Gender x Degree |
| Dairy |  |  |  |  |  |  |  |  |  |  |  |  |  |
| Olives, nuts, seeds | 1,2,3 |  |  |  |  | 3 |  |  |  |  |  |  |  |
| Herbs, spices, garlic, onions |  |  |  | 2 |  |  |  |  |  |  |  |  |  |
| Fruits |  |  |  |  |  |  |  |  |  |  |  |  |  |
| Vegetables |  |  |  |  |  |  |  |  |  |  |  |  | 1 |
| Olive oil |  |  | 2 |  |  |  |  |  |  |  |  |  | 1,2,3 |
| Bread, pasta, rice, other cereals | 1,2,3 |  |  |  |  | 1,2,3 | 1,2,3 | 1,2,3 |  |  | 1 |  |  |
| *Weekly* |  |  |  |  |  |  |  |  |  |  |  |  |  |
| Potatoes |  |  |  |  |  | 3 | 3 | 3 |  |  |  | 1,3 |  |
| Red meat and processed meat | 1,3 | 1,3 |  |  |  |  |  |  | 1 |  |  |  |  |
| Sweets |  |  |  |  | 1,2,3 | 1,3 |  |  |  |  |  |  |  |
| White meat |  |  |  | 1,2,3 |  |  |  |  |  |  |  | 1 |  |
| Fish. seafood |  |  |  |  |  |  |  |  |  |  |  |  |  |
| Eggs | 1,2,3 |  |  |  |  | 1,2,3 |  |  |  |  | 2 |  |  |
| Legumes | 1,3 |  |  |  |  |  |  | 1 |  |  |  | 1,2 |  |
| *Daily consumption of other food groups of interest* | | |  |  |  |  |  |  |  |  |  |  |  |
| Alcoholic drinks |  |  |  |  |  |  | 1,2,3 | 1 |  |  |  |  |  |
| Fast food | 1,2,3 | 1,2,3 |  |  |  |  |  |  |  |  |  |  |  |
| Precooked food |  |  |  |  |  |  | 3 |  | 1 |  |  |  |  |
| Note: All we considered significance when p<0.10. 1=significative in complete-case analysis; 2=significative in regression with imputed data (m=5); 3= significative in regression with imputed data (m=30). | | | | | | | | | | | | |  |

## Supplementary figures

Fig A. Correlation across independent variables


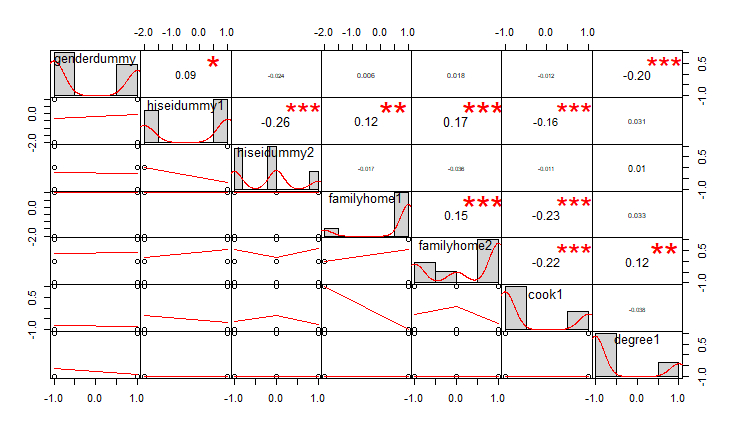


Note: genderdummy: Gender; hiseidummy1: SES (1); hiseidummy2: SES (2); familyhome1: Family home (1); familyhome2: Family home (2); cook1: CFHS; degree1: Degree

Fig B. Density plots of food groups after imputation of values: complete-case analysis and multiple imputation (m=5)


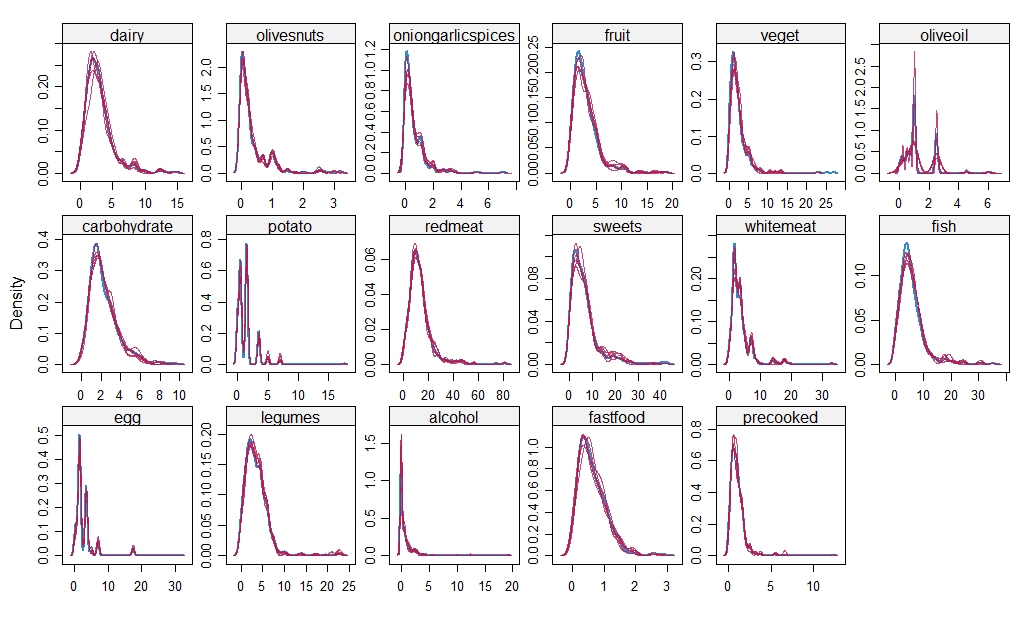


Note: blue colour: distribution of variables used in complete case-analysis; red colour: distribution of variables used after the imputation of values (m=5)

Fig C. Density plots of food groups after imputation of values: complete-case analysis and multiple imputation (m=30)


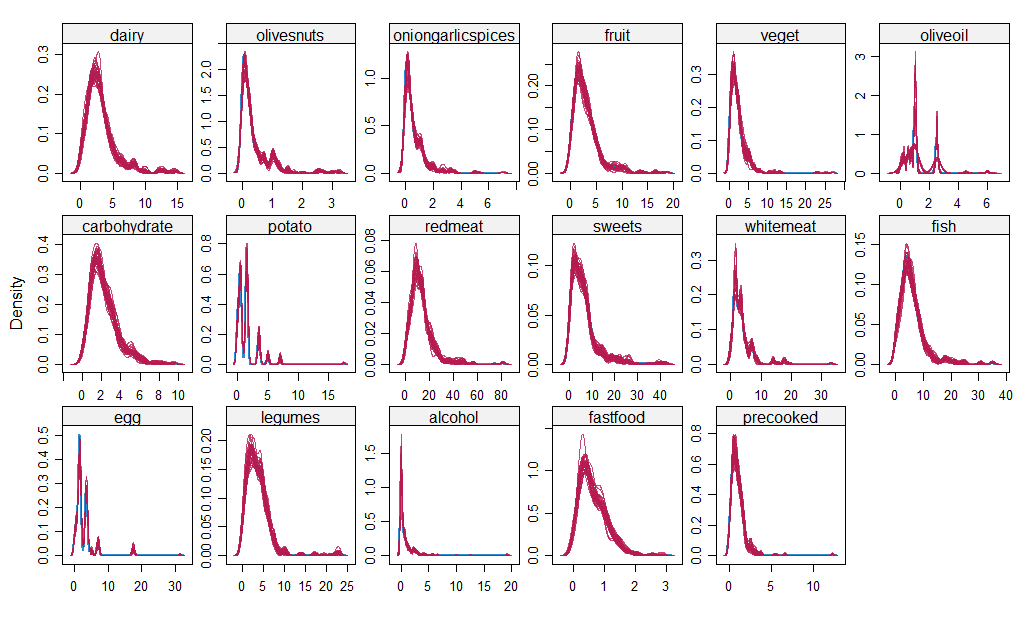


Note: blue colour: distribution of variables used in complete case-analysis; red colour: distribution of variables used after the imputation of values (m=30)

Fig D. Interaction effect between SES (1) and gender in the food group “Bread, pasta, rice, and other cereals” (*n=*593)


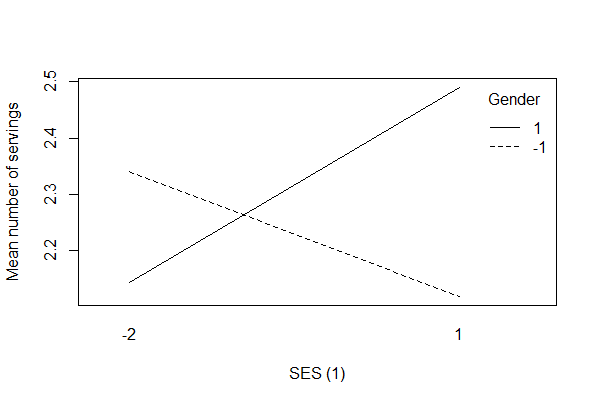


Fig E. Interaction effect between SES (1) and gender in the food group “Legumes” (*n=*593)


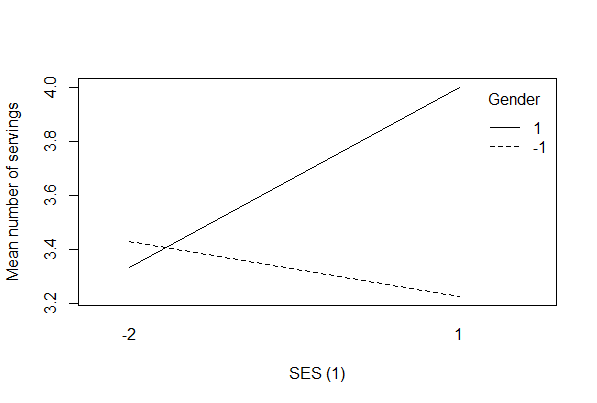


Fig F. Interaction effect between SES (1) and gender in the food group “Alcoholic drinks” (*n=*593)


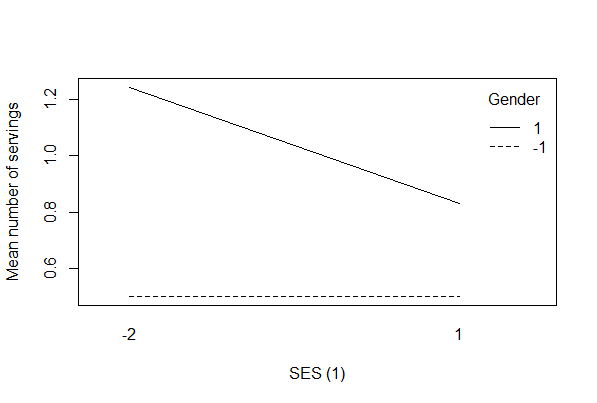


Note: The difference in the consumption of alcoholic drinks in women between low and high-medium socioeconomic status is practically zero. That is the reason why the slope is lower.

Fig G. Interaction effect between SES (2) and gender in the food group “Precooked” (*n=*593)


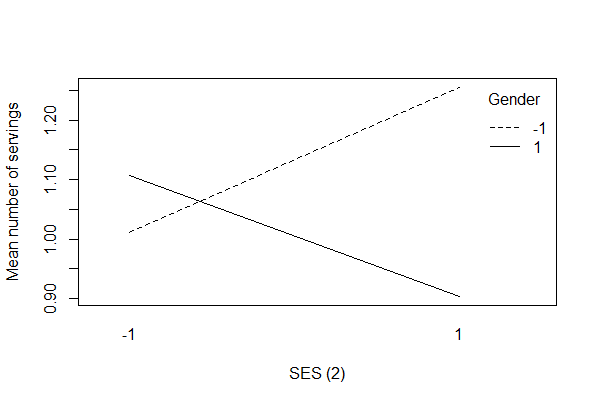


Fig H. Interaction effect between family home (1) and gender in the food group “Fast food” (*n=*593)


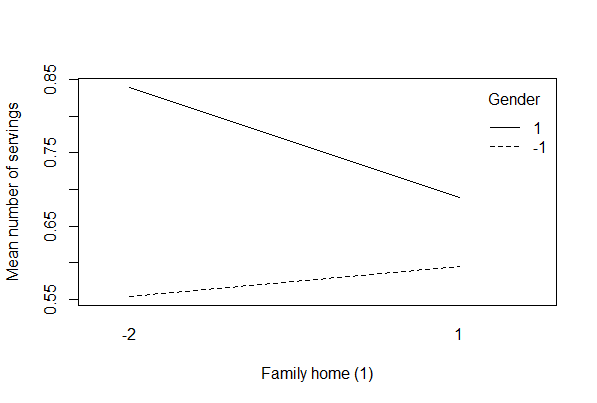


Fig I. Interaction effect between family home (2) and gender in the food group “Bread, pasta, rice, and other cereals” (*n=*593)


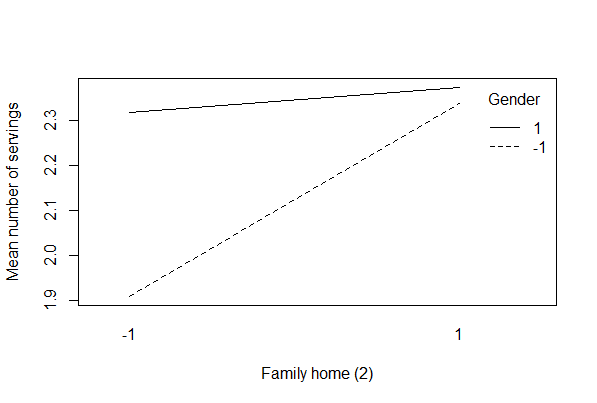


Fig J. Interaction effect between family home (2) and gender in the food group “Red meat and processed meat” (*n=*593)


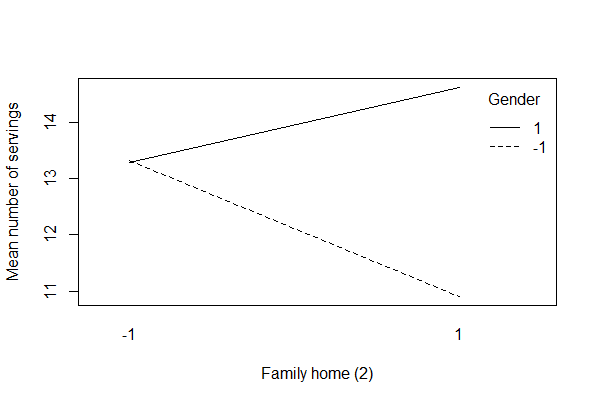


Fig K. Interaction effect between family home (2) and gender in the food group “Eggs” (*n=*593)


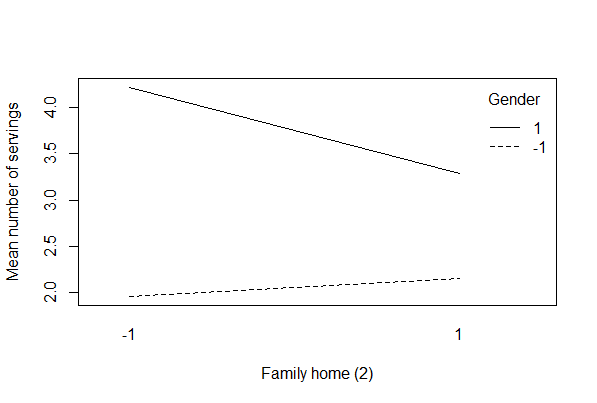


Fig L. Interaction effect between CFHS and gender in the food group “Potatoes” (*n=*593)


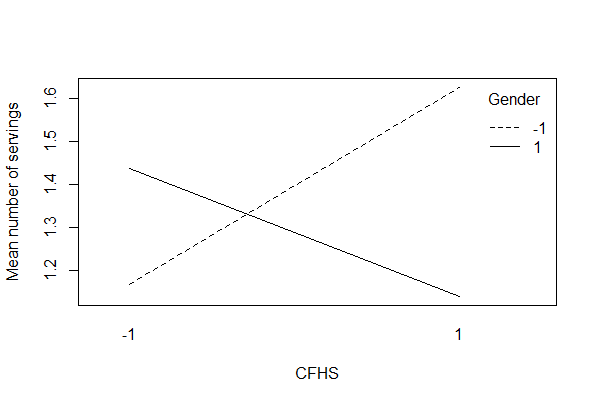


Fig M. Interaction effect between CFHS and gender in the food group “White meat” (*n=*593)


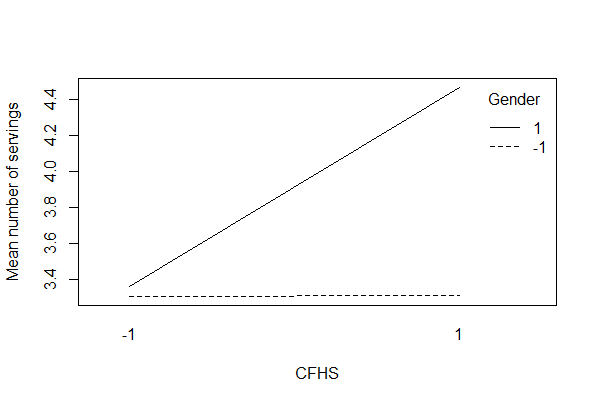


Note: The difference in the consumption of alcoholic drinks whether women cook for themselves or not is practically zero. That is the reason why the slope is lower.

Fig N. Interaction effect between CFHS and gender on “Legumes” food group (*n=*593)


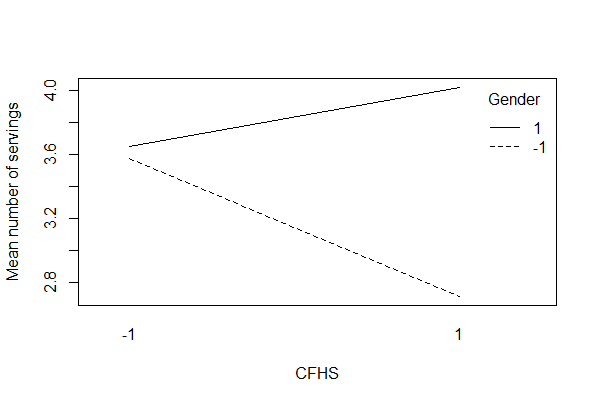


Fig O. Interaction effect between degree and gender on “Vegetables” food group (n=593)


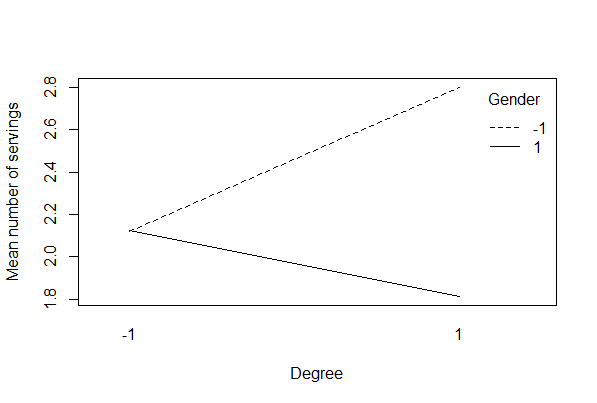


# References

Bach-Faig, A., Berry, E. M., Lairon, D., Reguant, J., Trichopoulou, A., Dernini, S., … Serra-Majem, L. (2011). Mediterranean diet pyramid today. Science and cultural updates. *Public Health Nutrition*, *14*(12A), 2274–2284. https://doi.org/10.1017/S1368980011002515

Sofi, F., Macchi, C., Abbate, R., Gensini, G. F., & Casini, A. (2013). Mediterranean diet and health status: an updated meta-analysis and a proposal for a literature-based adherence score. *Public Health Nutrition*, *17*(12), 2769–2782. https://doi.org/10.1017/S1368980013003169
