# Supplementary material for: Social determinants of food group consumption based on Mediterranean diet pyramid: A cross-sectional study of university students
Source: PLoS One. 2020 Jan 30;15(1):e0227620. doi: 10.1371/journal.pone.0227620 (PMC6992217; doi:10.1371/journal.pone.0227620)
Supplement: Attachment — Submitted filename: Response to Reviewers.docx [file pone.0227620.s002.docx]

**Rebuttal letter including responses to academic editor and reviewers**

Dear Editor,

Thank you for your comments and the opportunity to improve our paper, and we thank the reviewers for their work. We consider that the proposed changes improve the scientific quality of our manuscript. The changes have been highlighted. We have organized our response as follows. First, we have responded to the suggestions of the academic editor and the changes in the main manuscript have been highlighted in blue. Secondly, we have made the changes suggested by the journal (highlighted in blue in the main manuscript), and then we have made the corrections suggested by the reviewers (highlighted in yellow in the main manuscript).

# Responses to academic editor

*1. In your revised version, you need (…) add more background about differences in eating habits according to gender in the introduction.*

Thank you for your suggestion. We have added more background about differences in eating habits according to gender in the introduction. This can be seen on page 2 (lines 39-42).

*2. In addition, please note that the present submission is closely related to a previously published work by you: "Socioeconomic, demographic and lifestyle-related factors associated with unhealthy diet: a cross-sectional study of university students". In this regard, PLOS ONE criteria on related manuscripts require that related studies are adequately mentioned in the present submission (as you did), but PLOS ONE also require that the rationale of these separate analyses is clearly discussed, and the differences between the two works are clearly illustrated.*

Thank you for the observation. Please, if we misunderstood your comment, let us know.

The differences between our previous work entitled “Socioeconomic, demographic and lifestyle-related factors associated with unhealthy diet: a cross-sectional study of university students” and this new work are as follows. First, our previous article’s aim was to understand what factors were associated with unhealthy diet (defining this categorical variable by using an index of adherence to Mediterranean diet (1,2)) (as discrete variable) while the new work has used the recommendations of food group consumption based on the Mediterranean diet pyramid (3), grouping foods using this pyramid. This means that in our previous work we divided the sample, in some way, into two groups (unhealthy diet vs. healthy diet) and in the new paper we studied the level of compliance with the recommendations of Mediterranean diet. We wanted to show what food groups are a problem (or not) in our sample by separately studying food groups. An index is usually a good indicator and shows a general picture of the problem (in other words, you could know whether an individual gets a good or bad score), but it does not show in what food group you should improve public policies. In addition, we focused on social determinants, disregarding the individual characteristics of the university students. With our new work, we now know, for example, that students must improve vegetable consumption, red meat and processed meat consumption or alcoholic drink consumption.

Following your recommendations, we have changed the last paragraph of the Introduction, as can be seen on pages 3-4 (lines 64-75). In addition, we have also included some reflections related to these suggestions in the Discussion section (page 23, lines 343-346 and 348-350; pages 24-25, lines 381-391; page 25, lines 395-398; pages 25-26, lines 399-409).

1.	Sofi F, Macchi C, Abbate R, Gensini GF, Casini A. Mediterranean diet and health status: an updated meta-analysis and a proposal for a literature-based adherence score. Public Health Nutr [Internet]. 2013;17(12):2769–82. Available from: https://doi.org/10.1017/S1368980013003169

2.	Sofi F, Dinu M, Pagliai G, Marcucci R. Validation of a literature-based adherence score to Mediterranean diet: the MEDI-LITE score. Int J Food Sci Nutr [Internet]. 2017;68(6):757–62. Available from: http://dx.doi.org/10.1080/09637486.2017.1287884

3.	Bach-Faig A, Berry EM, Lairon D, Reguant J, Trichopoulou A, Dernini S, et al. Mediterranean diet pyramid today. Science and cultural updates. Public Health Nutr [Internet]. 2011;14(12A):2274–84. Available from: https://doi.org/10.1017/S1368980011002515

*3. If you would like to make changes to your financial disclosure, please include your updated statement in your cover letter.*

Thank you. We have changed our funding section on page 29 (lines 480-481).

# Journal requirements

*1. Please ensure that your manuscript meets PLOS ONE's style requirements, including those for file naming.*

We have adapted our paper to PLOS ONE’s style requirements. If you think we have made any mistakes in this adaptation, please let us know.

*2. Please include additional information regarding the survey or questionnaire used in the study and ensure that you have provided sufficient details that others could replicate the analyses. For instance, if you developed a questionnaire as part of this study and it is not under a copyright more restrictive than CC-BY, please include a copy, in both the original language and English, as Supporting Information.*

OK. We will attach the copy of the questionnaire in both languages under request.

*3. Please ensure that the findings of your previous analysis "Socioeconomic, demographic and lifestyle-related factors associated with unhealthy diet: a cross-sectional study of university students" are thoroughly discussed in the introduction and Discussion, to allow a broad overview of the field, and to illustrate the background of the present analysis.*

Following your suggestions, we have discussed the approach of both papers in the Introduction section (in the pages 3-4, lines 64-75), and in the Discussion section (page 23, lines 343-346 and 348-350; pages 24-25, lines 381-391; page 25, lines 395-398; pages 25-26, lines 399-409). Please, note that in the Discussion section some paragraphs are in yellow because they are also responses to Reviewer 1.

Please, if you have any further suggestions, let us know.

*4. We note that you have stated that you will provide repository information for your data at acceptance. Should your manuscript be accepted for publication, we will hold it until you provide the relevant accession numbers or DOIs necessary to access your data. If you wish to make changes to your Data Availability statement, please describe these changes in your cover letter and we will update your Data Availability statement to reflect the information you provide.*

OK. If PLOS ONE works with some repository, please let us know and we could upload the database in its repository.

*5. Please include captions for your Supporting Information files at the end of your manuscript, and update any in-text citations to match accordingly.*

OK. Following your suggestions and the Supporting Information guidelines, we have added the captions at the end of our manuscript. We have created a single file entitled “S1 Supporting information”, which includes all the supplementary analyses, tables and figures.

Please, if you consider that our supplementary material is not in line with the journal guidelines after the changes, let us know.

*6.* *We noted in your submission details that a portion of your manuscript may have been presented or published elsewhere.* *Please clarify whether this publication was peer-reviewed and formally published. If this work was previously peer-reviewed and published, in the cover letter please provide the reason that this work does not constitute dual publication and should be included in the current manuscript.*

Yes, the publication was peer-reviewed and formally published under the terms of the Creative Common Attribution 4.0. (<http://creativecommons.org/licenses/by/4.0/>). You can check this at the end of the following web page:

<https://bmcpublichealth.biomedcentral.com/articles/10.1186/s12889-018-6149-3>

For this reason, we can share and adapt our material for any purpose, if we give appropriate credit to it. In order to respect the CC-BY license, we have added the reference to our previous paper in the Figure 1 and in the main text.

Please, if you think we are mistaken, let us know. Another solution could be to refer the data cleaning process to our previous paper.

*7. While revising your submission, please upload your figure files to the Preflight Analysis and Conversion Engine (PACE) digital diagnostic tool, https://pacev2.apexcovantage.com/. PACE helps ensure that figures meet PLOS requirements. To use PACE, you must first register as a user. Registration is free. Then, login and navigate to the UPLOAD tab, where you will find detailed instructions on how to use the tool. If you encounter any issues or have any questions when using PACE, please email us at figures@plos.org. Please note that Supporting Information files do not need this step.*

Our figures meet PLOS requirements. If you need the files, please let us know.

# Review comments to the Author (Reviewer 1)

Thank you very much for your comments and suggestions. We think your review improves the quality of our manuscript and also clarifies some important points. All your suggestions are highlighted in yellow.

*1. Introduction: You should include more background on risk behaviors in students that trigger an unhealthy diet, e.g. skipping meals frequently, eating between meals and having a high consumption of ultra-processed food.*

Following your suggestion, we have included more background about risk behaviours in students in page 3 (lines 58-61).

*2. Methods*

*i. Participants: It is important to mention that it is a non-probabilistic sample, for convenience instead of a representative sample.*

Thank you. Following your advice, we have added the required information on page 4 (lines 84-85).

*ii. According to several studies, the importance of the degree course variable (Health Sciences, Social Sciences) has been communicated, together with its conclusion in its article: “Socioeconomic, demographic and lifestyle-related factors associated with unhealthy diet”, BMC Public Health 2018, that said: “…. finally, not studying a health-related course are the factors associated with a lower quality diet ”. This variable must be included in your analyzes.*

Thank you for your suggestion. Following your recommendation, we have added the variable “degree course”. A paragraph was added in Methods section (page 6, lines 135-138), Results section (pages 20-21, lines 278-291) and Discussion section (pages 24-25, lines 380-392). We have changed “Table S5. Independent variables: dummy coding”, and Table 1 adding the new information. We have created Table 6. The results of the regression analysis have also changed.

Please, if you have any further suggestions, let us know.

*iii. Why is it mentioned that 141 foods were divided into 12 groups in the FFQ? And then, in the analysis of the data (tables) 17 food groups appear. This must be clarified.*

Thank you. This was unclear. We have revised this point and changed the text on page 5 (lines 98-99) and we have modified Table S1.

*iv. The analysis shown in Table 3 and 4 about mean differences in food group consumption by family´s socioeconomic status or the location of the family home, respectively. They cannot present two independent analyzes for the same variable; this increases the type 1 error. Therefore, I would suggest you to work the three respective levels for each variable, despite the fact that in the multiple regression you worked with the dummies variables. You should perform also ANOVA with multiple comparisons in case you show significant differences.*

Thank you. Following your suggestion, we have performed the ANOVA analysis with the three respective levels for each variable. The changes have been introduced in Table 3 and Table 4, and the related results rewritten.

*v. The table 7 shows multiple regression analysis of food groups, social determinants and interaction. More precision is lacking in the final model, considering the interactions that were significant. I suggest you to generate different models for the combinations of significant interactions, with this you can really respond to the second objective of your paper that says: “we analyze how these social determinants and the interaction with gender may affect different food group consumption.”*

Thank you for your comment. Following your suggestion, we have added a new table (Table 9) including this. This table is entitled “Multiple regression analysis: fitted models for significant interactions”. The analysis was performed including all the significant interactions and significant independent variables.

However, if we have misunderstood your suggestion, please, let us know.

*3. Discussion*

*i. In relation with the limitations of the study, you should mention that the final sample only represented 4% (593 / 15,278) of the population.*

Thank you, we have added the suggested information. We also added this because we were using multiple imputation techniques, working with 924 students. You can find that on page 26 (lines 415-417).

*ii. In addition, there is no clarity of the level that students take, because it is not the same reality to be a freshman in comparison with those of higher courses.*

Thank you. Following your recommendation, we have written this information as a limitation of the study (page 26, lines 417-419).

# Review comments to the Author (Reviewer 2)

Thank you very much for your comments and review.
