# Supplementary material for: Social determinants of food group consumption based on Mediterranean diet pyramid: A cross-sectional study of university students
Source: PLoS One. 2020 Jan 30;15(1):e0227620. doi: 10.1371/journal.pone.0227620 (PMC6992217; doi:10.1371/journal.pone.0227620)
Supplement: Attachment — Submitted filename: Response to Reviewers.docx [file pone.0227620.s003.docx]

**Rebuttal letter including responses to academic editor and reviewers**

Dear Academic Editor,

Thank you for your comments and the opportunity to improve our paper, and we thank the reviewers for their work, once again. The changes have been highlighted.

On this occasion, we have responded to your suggestion divided into three paragraphs and the changes in the main manuscript have been highlighted in yellow.

# Responses to academic editor

1. “*Your manuscrip submited to Plos One have valuable information, which was not at all covered in the previous submission; and the Result section seems to contain very interesting data.”*

Thank you very much.

2. *“However, in my opinion and on the basis of the opinion of one of the Reviewers, the difference between this manuscript and your previously published manuscript is not well illustrated in the Introduction and in the Discussion (in particular, the first paragraph of the discussion in which it seems to mix together the two studies). Therefore, you need to provide a better rationale for this study, and carefully explain in the Discussion (and not only in your response to reviewers) what insights were found by your new analysis, without conflating results that were already presented in the previous manuscript.”*

Thank you for your suggestion.

We have simplified the first paragraph of the Discussion section in order to improve the precision and it now read (page 23, line 329-330): “*This study had two main objectives, which we addressed by means of two analyses. First, we studied the level of compliance* (…)”. On the other hand, in the Discussion section (page 26, lines 416-424), we have added an explanation which illustrates the difference between our previous work and this new one. It now reads as follows (we have underlined the explanation): “*The previous work [14] aimed to analyse the association between the individual and social characteristics of the sample and quality of diet, categorising an index of adherence to the Mediterranean diet as healthy/unhealthy diet [36,68]. We decided to adopt a different approach in this work because the previous study presented a knowledge gap that we wish to fill. Despite an index usually being a good indicator and showing a general picture of the food habits in the study sample through a global score, it does not clearly show in what food groups decision makers should improve public policies. The previous work studied individual characteristics of the sample (such body mass index), but in this work we focus on social determinants, disregarding the former. For this reason, we followed a different approach in order to show which food groups pose (or not) a problem in the pursuit of a healthier dietary pattern, which could improve long-term health*”.

In addition, we added a new paragraph in the Discussion section (page 27, lines 429-441) trying to show the insights of our work and suggesting recommendations to decision/policy makers: “*Following our results, policy makers should make an effort to promote the Mediterranean diet among university population due to its many benefits [19,20], as students do not fully comply with the recommendations on different food groups. In addition, they should to address the gender gap in the consumption of unhealthy foods, such as sweets, alcoholic drinks and fast food (men showed a higher consumption of these groups), and in healthy foods, such as vegetables or fruits, where men showed a lower consumption, but legumes consumption is higher among men with a better socioeconomic position and who also cook for themselves. Regarding other social determinants, knowledge of healthy food habits should be improved among Social Science students considering the interaction with gender. Despite our results not showing a substantial association with socioeconomic position, it should be considered since previous literature has shown the influence of socioeconomic status on food habits. In addition, and concerning family home, policy efforts may not be necessary*.”

Please, if you have any other suggestion or you think we should improve precision, let us know.

3. *“Moreover, the introduction needs to make clear that the same sample was analysed in a previous article, and that the data of Table 1 (where the characteristics of the study sample are reported) have been previously shown*.”

We have rewritten a line in the last paragraph of the Introduction section trying to make clear that we use the same database. It now reads (page 4, lines 79-82): *“**This work uses a sample of students that was used in an earlier work aiming to study the factors associated with an unhealthy diet [14]. That study analysed diet quality through the use of an index, while the current work has adopted a different approach, using new variables.”* Please, if you think we can explain this better, let us know.

On the other hand, and regarding Table 1, we have added a line in the Results section. This now reads (page 9, lines 183-189): *“Table 1 shows the characteristics of the population by student gender. The students’ age, whether they cooked (or not) for themselves, and the degree course were previously shown in our earlier work [14]. In the present study, we also included the location of the family home, but using three categories that consider the size of the family home town. The socioeconomic status of the family is a new variable.”*. Please, if you think we can write or express this issue in a better way, let us know.
